# Herpes simplex virus diverts CIN85 endosomal cargo for exocytosis to evade antiviral responses: a novel role for the viral immediate-early protein ICP0

**DOI:** 10.1128/mbio.02143-25

**Published:** 2025-09-24

**Authors:** Hope Waisner, Rabina Saud, Sarah Lasnier, Sreenath Muraleedharan Suma, Kim Foster-Lemieur, Maria Kalamvoki

**Affiliations:** 1 Department of Microbiology, Molecular Genetics, and Immunology, University of Kansas Medical Center 21638, Kansas City, Kansas, USA; Princeton University, Princeton, New Jersey, USA

**Keywords:** HSV-1, CIN85, ICP0, endosomes, extracellular vesicles, autophagy, immunoevasion, innate immunity

## Abstract

Endocytosis and exocytosis pathways are critical for the survival of the cells. Viruses often hijack these trafficking pathways to support cell entry, replication, and egress and to evade host antiviral responses. The infected cell protein 0 (ICP0) is an immediate early protein of alphaherpesviruses also known as the promiscuous transactivator. Most of the ICP0 functions are performed in the nucleus; however, ICP0 translocates to the cytoplasm after enabling viral gene expression where very little is known about its cytoplasmic roles. ICP0 interacts with the Cbl-interacting protein of 85 kDa (CIN85), a scaffolding protein for factors involved in endocytosis and protein sorting. The ICP0/CIN85 complex promotes endocytosis of surface receptors, including the epidermal growth factor receptor (EGFR) to suppress signaling and the virus entry receptor nectin-1 to ensure virus spread in uninfected cells. CIN85 recognizes consensus motifs Px(P/A)xxR in its binding partners, and two such motifs are stretched between ICP0 246–258 aa. Deletion of the CIN85-binding motifs of ICP0 disrupts its interaction with CIN85 and the localization of ICP0 to CIN85 vesicle-like structures. This ICP0 deletion further disrupts exocytosis of cargo associated with the CIN85 structures through extracellular vesicles (EVs), including autophagy and innate immune factors. Consequently, this ICP0 mutant virus fails to counteract antiviral responses and displays decreased progeny virus production. Overall, these studies have uncovered a novel mechanism by which HSV evades host antiviral responses through subjugation of endosomal and exocytosis pathways.

**IMPORTANCE:**

Herpes simplex virus persists lifelong. For a successful infection, the virus has evolved different mechanisms, often complementary and redundant, to evade host defense pathways, highlighting that viral proteins are multifunctional. Multifunctional proteins display a variety of features, including different localization patterns, post-translational modifications, and ability to interact with different factors. The infected cell protein 0 (ICP0) of the virus encompasses three main functions; it is a promiscuous transactivator, an E3 ubiquitin ligase, and an inhibitor of DNA repressor complexes. The protein localizes to the nucleus early in infection and translocates to the cytoplasm following virus replication. In the cytoplasm, ICP0 interacts with the Cbl-interacting protein of 85 kDa (CIN85), an endocytosis adaptor, promoting surface receptor endocytosis, cargo sorting, and exocytosis. Innate immunity and autophagy-related factors are also found to be exocytosed via this pathway. This likely represents a novel immunoevasion function of HSV-1 ICP0 to suppress antiviral signaling.

## INTRODUCTION

To successfully invade the host, HSV-1 must overcome strong antiviral responses, and infected cell protein 0 (ICP0) plays a fundamental role in this process. ICP0 localizes in the nucleus at discrete interchromosomal accumulations of proteins, known as nuclear domain 10 (ND10) bodies (1–9). The genome of HSV-1 is in association with ND10s, where it is transcriptionally repressed. ICP0 has an E3 Ub ligase activity that is attributed to its N-terminal zinc-binding RING finger domain (RF) (aa 116–156) and is conserved among α-herpesvirus ICP0 orthologues. ICP0 degrades essential components of ND10s, including the promyelocytic leukemia protein (PML) and the Speckled 100 kDa protein (Sp100), and disperses cellular repressors to viral DNA, activating transcription (3, 4, 7–17). Failure of ICP0 to execute these functions impairs virus replication.

Late gene expression triggers translocation of ICP0 to the cytoplasm. Although the exact roles of ICP0 in the cytoplasm remain largely elusive, it is known to interact with the Cbl-interacting protein of 85 kDa (CIN85). CIN85 belongs to a family of adaptor proteins with established roles in the spatial and temporal assembly of protein complexes during receptor endocytosis (18–21). The protein contains three N-terminal SH3 domains, followed by a central proline-rich region and a C-terminal coiled-coil domain. Via its SH3 domains, CIN85 recognizes a consensus Px(P/A)xxR motif present in several proteins involved in endocytosis, including the Cbl E3 ligase that ubiquitinates surface receptors such as cytokine and chemokine receptors, promoting their internalization; synaptojanin, a brain-enriched lipid enzyme involved in membrane trafficking at the synapse; Alix, an accessory molecule of the endosomal sorting complexes required for transport (ESCRT), as well as regulators of the actin cytoskeleton such as cortactin. These interactions suggest that CIN85 is a central adaptor, involved in assembly of endocytic machineries for surface receptor internalization and suppression of surface receptor signaling.

ICP0 has four putative CIN85 SH3 binding motifs, three of which were found between the aa 245–395 (22). The GST-fusion form of ICP0 (245–395 aa) can pull down CIN85 from cell lysates, and this interaction promotes downregulation of total and surface levels of the epidermal growth factor receptor (EGFR) in the absence of EGF ligand (22). Thus, it was proposed that ICP0, along with CIN85 and Cbl, negatively regulate receptor tyrosine kinases (RTKs) (22). Considering that RTKs have roles in host defense, inflammation, cell survival, and autoimmunity, the virus-induced reduction of their surface abundance could serve as an immunoevasion mechanism (23–27). The ICP0/CIN85/Cbl complex also promotes reduction of the virus entry receptor nectin-1 from the surface of infected cells to increase its probability of entering uninfected cells (28). Overall, HSV-1 infected cells likely modulate endocytosis processes via ICP0/CIN85 interaction.

Previous studies from our laboratory have found that infection with HSV-1(F) increases the release of CD63+ extracellular vesicles (EVs) (29–32). These EVs exhibit antiviral properties, in part, due to the incorporation of the stimulator of interferon genes (STING) protein (30, 32). More recently, we found that infection with two mutant viruses that are unable to efficiently counteract autophagy, the ΔICP34.5 and ΔICP0 viruses, has substantially different effects on exocytosis (32, 33). Infection with the ΔICP34.5 virus leads to increased production of EVs from the plasma membrane that contain markers of microvesicles and apoptotic bodies. In contrast, infection with the ΔICP0 virus did not result in greater EV production although the EVs that were produced were characteristically similar to those released by cells infected by the wild-type virus (33). Interestingly, CIN85 was found to be released in EVs from wild-type virus-infected cells, but not from cells infected with the ΔICP0 virus. In cells infected with the ΔICP34.5 virus, CIN85 was released to a lesser extent (33). While CIN85 appeared to be degraded in ΔICP34.5-infected cells, this may not be the case in ΔICP0-infected cells (33). These findings indicate that ICP0 plays a role in EV production during HSV-1 infection and facilitates CIN85 release in these vesicles. Given all these findings, we aimed to further characterize the interplay between CIN85 with ICP0 and its impact on endocytosis, autophagy, and exocytosis during HSV-1 infection, as well as its potential role in overall viral evasion.

Here, we report that ICP0 and CIN85 colocalize on the surface of cytoplasmic vesicular structures. To investigate the significance of their interaction, we developed an ICP0 mutant virus (ICP0 delta 244–277) with a deletion of amino acids 244–277, which removed two consecutive CIN85-binding motifs. This mutation decreased ICP0’s ability to interact with CIN85 and prevented its localization to the CIN85 structures. Although the E3 ubiquitin ligase activity of ICP0 remained intact with this deletion, the mutant virus failed to efficiently counteract antiviral responses and displayed decreased progeny virus production. Further characterization of the CIN85 structures revealed a chimeric phenotype, containing markers of multiple intracellular pathways, including early endosomes (Rab5), late endosomes (Rab7), and autophagosomes (autophagy-related factor ATG5, autophagosome biogenesis factor LC3-B, and autophagy adaptor p62/SQSTM1) (34–38). Notably, HSV-1 could block autophagy in cells where these chimeric ICP0/CIN85 structures were present, indicating that they were not autophagosomes. We also discovered that a subset of cargo which associated with the ICP0/CIN85 structures, including CIN85 itself, was exocytosed through EVs. Unlike CIN85, ICP0 exocytosis was not observed. In cells infected with the ICP0 delta 244–277 virus, exocytosis of various cargo proteins—including EGFR, Sp100, LC3-B, and p62/SQSTM1—was impaired. This likely represents a novel immune evasion role of HSV-1 ICP0 that involves subjugation of endocytosis and other sorting pathways to regulate cargo exocytosis and/or degradation.

## RESULTS

### Deletion of two CIN85-binding domains of ICP0 disrupts its interaction with CIN85 and abrogates its localization to CIN85 structures, resulting in impaired virus growth

**Fig 1:**
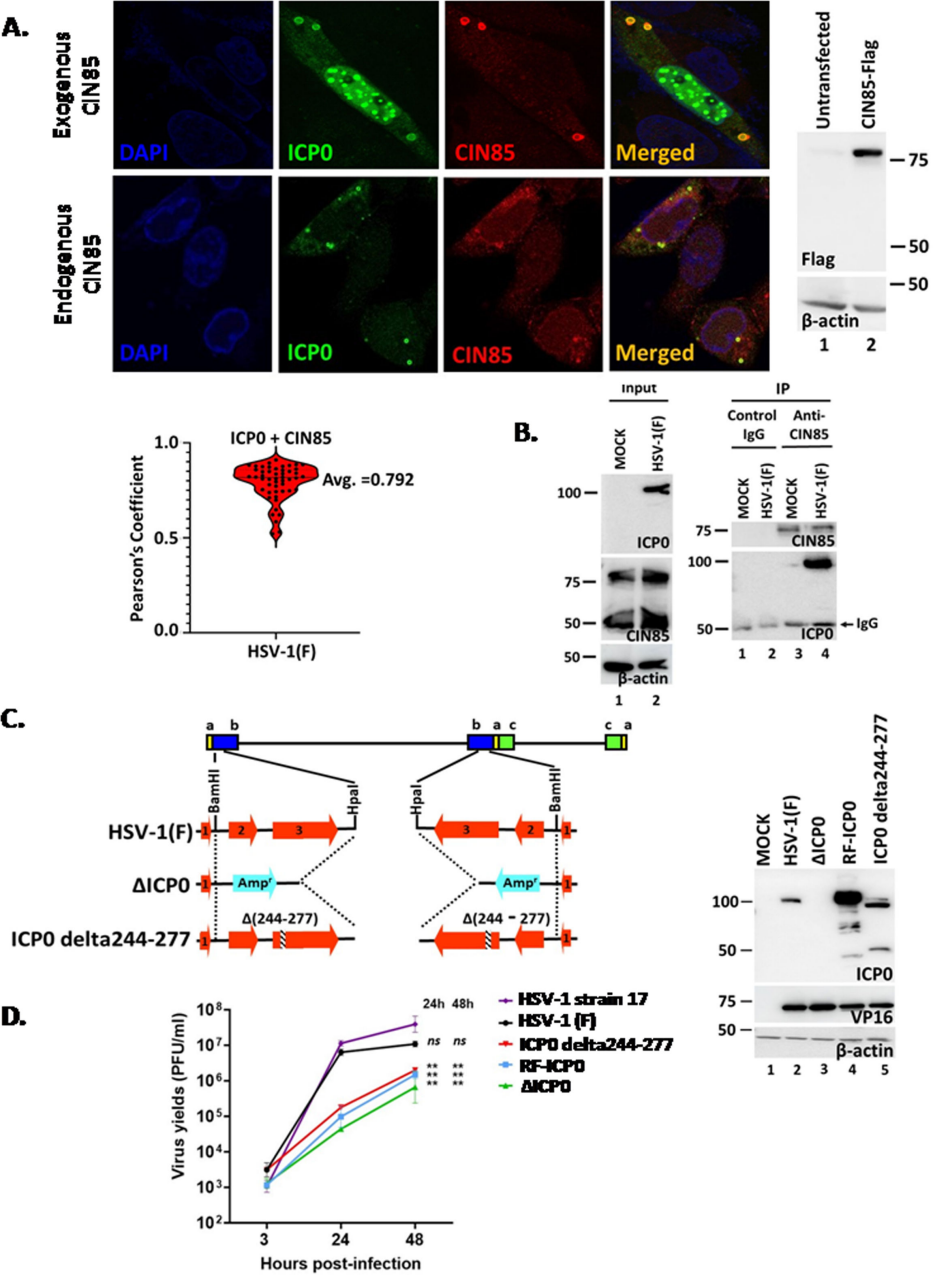
Development of an HSV-1 mutant virus with a deletion of the ICP0 amino acids 244–277. (**A**) HEp-2 cells were transfected with a CIN85-Flag-expressing plasmid for 24 h, followed by HSV-1(F) infection (10 PFU/cell). Cells were fixed at 14 h post-infection and doubly reacted with ICP0 and Flag antibodies. Images were captured using a Leica TCS SP8 STED microscope. Western blot analysis of exogenous CIN85-Flag is also depicted. In the lower panel, HEp-2 cells, not transfected, were infected with HSV-1(F) (10 PFU/cell). The cells were fixed at 14 h post-infection and doubly reacted with anti-ICP0 and anti-CIN85 antibodies. Images were acquired as above. The Pearson’s correlation coefficient for the ICP0/CIN85 colocalization was measured for the vesicular structures found in 50 cells using ImageJ. (**B**) HEL cells were infected with HSV-1(F) (1 PFU/cell) or remained uninfected. The cells were harvested at 14 h post-infection, mildly lysed, and equal amounts of proteins from total cell lysates were immunoprecipitated with a rabbit anti-CIN85 antibody or a rabbit isotype control IgG. Immunoprecipitated proteins were analyzed with a mouse ICP0 or a mouse CIN85 antibody. One-fifth of the input proteins was analyzed for ICP0, CIN85, while β-actin served as a loading control. (**C**) A virus with an ICP0 244–277 aa deletion was developed as described in Materials and Methods. U2OS cells were infected with the WT virus and various ICP0 mutants (10 PFU/cell). The cells were harvested at 14 h post-infection, and equal amounts of proteins from total cell lysates were analyzed for ICP0 and VP16. β-Actin served as a loading control. (**D**) HEL cells were infected with HSV-1(F), HSV-1 strain 17, ICP0 delta244-277, RF-ICP0, and ΔICP0 (0.01 PFU/cell). The cells were harvested at 3, 24, and 48 h post-infection, and progeny virus production was quantified by plaque assays.

Three putative CIN85-binding motifs within the 245–395 aa region of ICP0 mediate its binding to CIN85 (22). To assess whether ICP0 and CIN85 colocalize, we transfected HEp-2 cells with a CIN85-Flag expression plasmid and then infected them with HSV-1(F). Immunofluorescence analyses revealed that ICP0 and CIN85 colocalize in vesicle-like structures within the cytoplasm of infected cells (Fig. 1A). The Pearson’s correlation coefficient was calculated for all ICP0/CIN85 vesicle-like structures from 50 cells and was found to average at 0.792, supporting the colocalization of ICP0 with CIN85 (Fig. 1A). This colocalization was confirmed during infection of untransfected cells when endogenous CIN85 was present (Fig. 1A). As further evidence that ICP0 and CIN85 interact, we performed an immunoprecipitation (IP) assay (Fig. 1B). hTERT-immortalized human embryonic lung fibroblasts (hTERT-HEL) were either infected with HSV-1(F) (1 PFU/cell) or left uninfected. At 14 h post-infection, cells were harvested, lysed, and subjected to immunoprecipitation using either a CIN85 antibody or an isotype IgG control. ICP0 was detected only in HSV-1 infected samples where the anti-CIN85 antibody was used, confirming the interaction between ICP0 and CIN85 (Fig. 1B).

Two of the putative CIN85-binding domains of ICP0 are located within the 244–258 amino acid region (22). To investigate their role, we developed a mutant virus carrying the 244–277 aa deletion in both copies of ICP0 using a ΔICP0 BAC system, which contains an ampicillin open reading frame in ICP0 locus, as has been previously described (Fig. 1C) (7, 8). The ICP0 deletion was verified by sequencing, and the expression of several viral genes, including the truncated ICP0, gC, and VP16, was confirmed by western blot (Fig. 1C and 4B). To assess the impact of this deletion on viral replication, we compared the growth of the ICP0 244–277 aa deletion mutant with its parental virus HSV-1(F), as well as an ICP0-null virus (ΔICP0), an ICP0 E3 ubiquitin ligase mutant (RF-ICP0), which carries C116A and C156A substitutions in the RING finger domain of ICP0 (RF-ICP0), and its parental virus HSV-1 strain 17 by performing low multiplicity infections (0.01 PFU/cell). We found that all ICP0 mutants displayed an approximately 200-fold decrease in progeny virus production compared to both wild-type virus strains (Fig. 1D). Since the deletion in ICP0 is proximal to the RING finger domain, which is essential for counteracting antiviral responses, we examined whether this mutation affected ICP0’s E3 ubiquitin ligase activity. To test this, we performed a kinetic analysis to compare the degradation of Sp100, a known ICP0 substrate, in cells infected with the WT virus, the RF-ICP0 mutant, and the new ICP0 mutant at 5 and 10 h post-infection (2, 13). Our results showed that the ICP0 244–277 aa deletion mutant degraded Sp100 protein similar to the WT virus (Fig. S1A). We also monitored the dispersion of the ND10 bodies by analyzing the nuclear localization of Sp100 and ICP0. By 4 h post-infection, a comparable number of cells displayed dispersed ND10 bodies and diffuse nuclear ICP0 (Fig. S1B). By 8 h post-infection, a comparable number of infected cells displayed cytoplasmic ICP0 (Fig. S1B). Quantification of cells with nuclear vs cytoplasmic ICP0 at 2, 4, and 8 h after infection with the WT virus or the ICP0 delta244–277 mutant shows that the two viruses have similar kinetics for nuclear and cytoplasmic ICP0 accumulation (Fig. S1C). Together, these findings indicate that the E3 ubiquitin ligase activity of the novel ICP0 mutant (244–277) remains intact.

**Fig 2:**
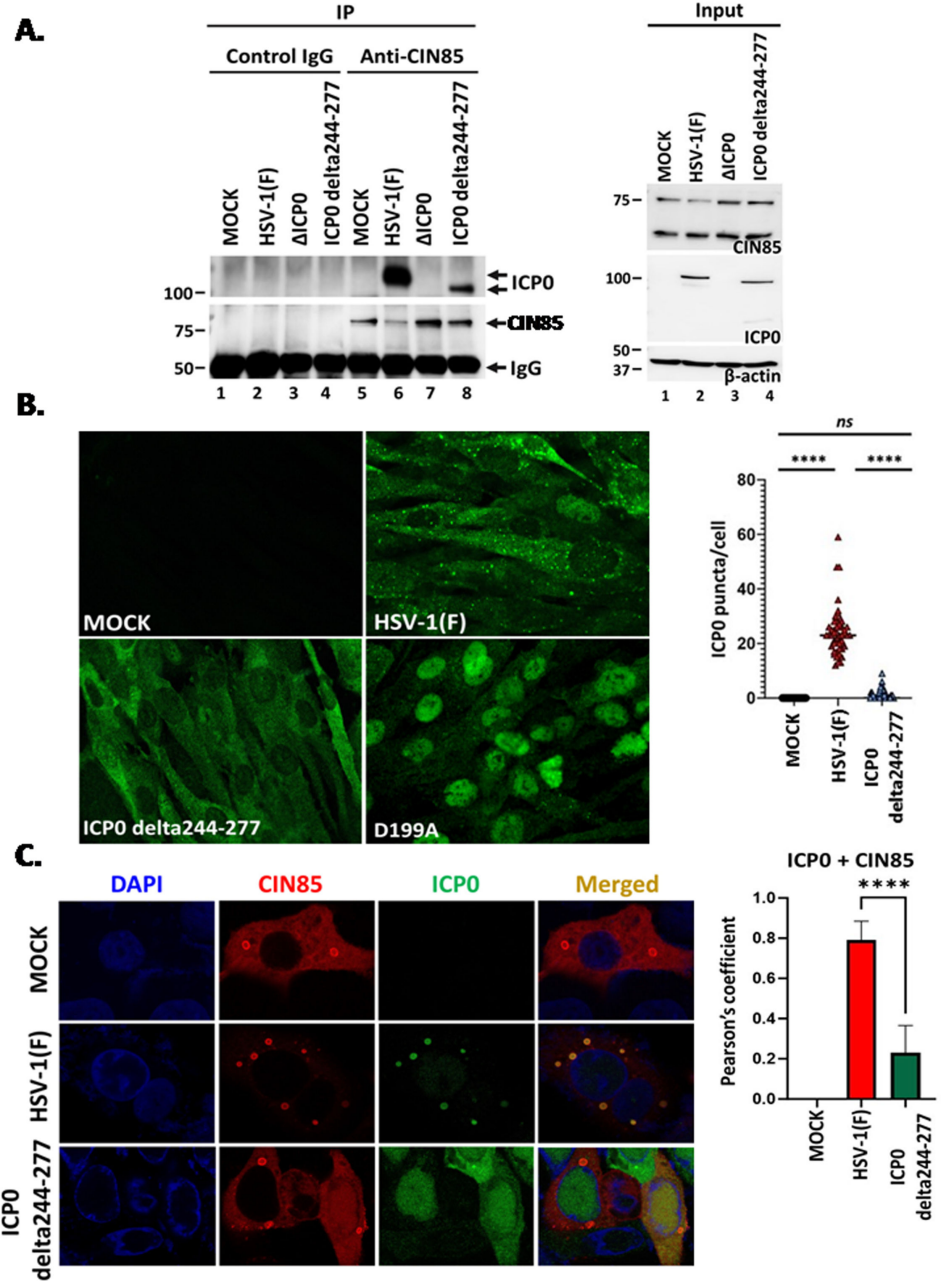
Deletion of the ICP0 244–277 aa abrogated ICP0/CIN85 interaction and the localization of ICP0 to the CIN85 structures. (**A**) hTERT-HEL cells were infected with HSV-1(F), ΔICP0, and ICP0 delta244–277 virus (1 PFU/cell) or remained uninfected. The cells were harvested at 14 h post-infection, and equal amounts of proteins from total cell lysates were immunoprecipitated with either a rabbit anti-CIN85 antibody or an isotype control IgG. Immunoprecipitated proteins were analyzed with a mouse ICP0 and a mouse CIN85 antibody. The 1/5 of the input proteins was analyzed for ICP0 and CIN85, while β-actin was used as a loading control. (**B**) hTERT-HEL cells were infected with HSV-1(F), ICP0 delta244-277, or D199A virus (10 PFU/cell). The cells were fixed at 14 h post-infection and stained with an ICP0 antibody. Images were captured using a Leica TCS SP8 STED microscope. The average number of ICP0 puncta per cell was estimated after analyzing over 50 cells with cytoplasmic ICP0. (**C**) Vero cells were transfected for 24 h with the CIN85-Flag-expressing plasmid and infected with the WT virus, the ICP0 delta244–277 mutant (10 PFU/cell), or remained uninfected. The cells were fixed at 14 h post-infection and doubly reacted with an anti-CIN85 and an anti-ICP0 antibody. Images were captured as above, and colocalization of ICP0 with CIN85 was calculated with Pearson’s correlation coefficient using ImageJ. ****P < 0.0001.

In the next series of experiments, we analyzed how the ICP0 244–277 aa deletion affects ICP0 binding to CIN85 and its localization to the CIN85 structures. Replicate cultures of hTERT-HEL cells were infected with HSV-1(F), ΔICP0 virus, and the ICP0 244–277 aa deletion mutant or left uninfected. At 14 h post-infection, immunoprecipitations were performed using either a CIN85 antibody or an isotype IgG control, and immunoprecipitated ICP0 was detected by western blot. As shown in Fig. 2A, CIN85 interacted with wild-type ICP0 but displayed weak binding to the mutant ICP0. This weak binding is likely due to the two remaining CIN85 binding motifs in ICP0. When we analyzed the localization of the new ICP0 mutant at a late time post-infection, we found that it displayed a diffuse pattern within the cytoplasm and did not form puncta structures like the WT ICP0 (Fig. 2B). This phenotype persisted until the endpoint of the infection (data not shown). On average, WT virus-infected cells displayed approximately 25 ICP0 puncta per cell, whereas cells infected with the ICP0 (244–277 aa) mutant displayed fewer than one punctum per cell (Fig. 2B). As an additional control, we infected cells with an ICP0 mutant virus carrying an aspartic acid to alanine substitution at position 199 (D199A), which prevents ICP0 from exiting the nucleus. As shown in Fig. 2B, no ICP0 puncta were observed in D199A-infected cells in contrast to WT virus-infected cells (39). Finally, we determined that CIN85 localizes to vesicular structures in ICP0 delta244–277 virus-infected cells that are morphologically similar to the CIN85 structures formed in WT virus-infected or uninfected cells (Fig. 2C). We also noticed that approximately 10% of the cells in ICP0 delta244–277 infected cultures contained CIN85 in the nucleus. Nuclear localization of a sumoylated form of CIN85 has been reported, but its function is unknown (40). Perhaps, it is involved in cytokinesis like several ESCRT components (41). These findings indicate that the 244–277 aa region of ICP0 is essential for ICP0 localization to the CIN85 vesicles following the initiation of virus replication and for optimal virus yields.

### The ICP0/CIN85 structures are chimeric and contain markers of early and late endosomes and autophagosomes

**Fig 3:**
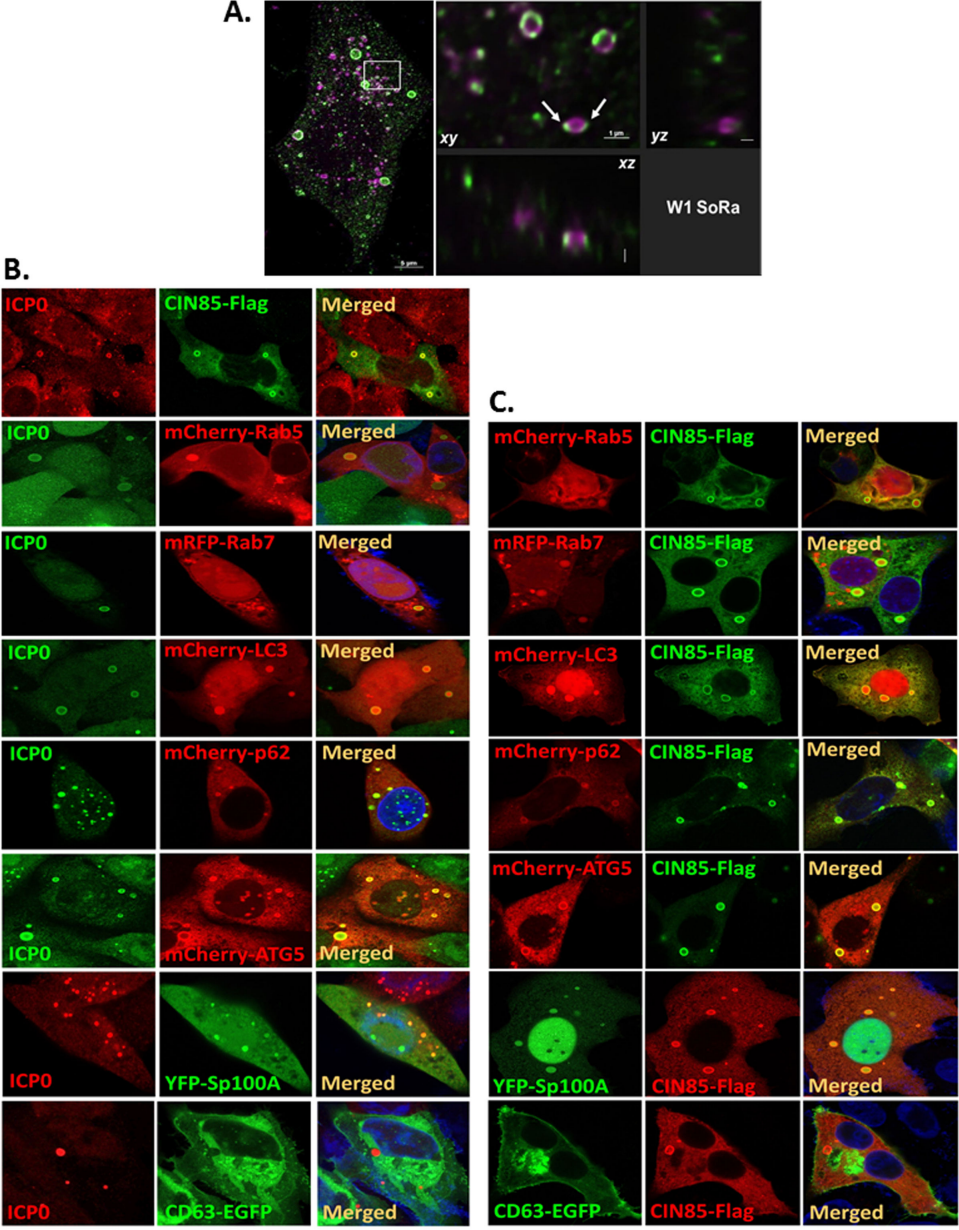
ICP0 localizes on the surface of the CIN85 structures, along with early and late endosome, autophagy, and innate immunity factors. (**A**) Vero cells were transfected with a CIN85-Flag expressing plasmid for 24 h followed by HSV-1(F) infection (10 PFU/cell). Cells were fixed at 14 h post-infection and stained with an ICP0 and a Flag antibody. Images were captured using a CSU-W1 SoRa confocal microscope. (**B and C**) Vero cells were transfected with plasmids expressing CIN85-Flag, mCherry-Rab5, mRFP-Rab7, mCherry-LC3, mCherry-p62, mCherry-ATG5, YFP-Sp100A, and CD63-EGFP or co-transfected with a CIN85-Flag-expressing plasmid and plasmids expressing the above-mentioned proteins. At 24 h post-transfection, the cells were infected with HSV-1(F) (10 PFU/cell). Cells were fixed at 14 h post-infection and probed with an ICP0 or a Flag antibody. Images were captured using a TCS SP8 STED microscope.

We next sought to characterize the ICP0/CIN85 cytoplasmic puncta structures. Vero cells were transfected with a CIN85-Flag-expressing plasmid, infected with HSV-1(F), and processed for immunofluorescence analysis. Captured images, using a CSU-W1 SoRa super resolution confocal microscope, revealed that both ICP0 and CIN85 localized to the surface of vesicular structures (Fig. 3A).

We next used a targeted approach to further characterize the proteome of the CIN85 structures where ICP0 localizes. Vero cells were either co-transfected with plasmids expressing markers of various vesicular compartments along with a CIN85-Flag-expressing plasmid or transfected with individual plasmids followed by HSV-1(F) infection. Uninfected transfected cells served as controls. We found that, in addition to CIN85, ICP0 structures were associated with the early endosomal marker Rab5 and the late endosomal marker Rab7 (Fig. 3B). Furthermore, ICP0 colocalized with certain autophagosome markers, including ATG5, an autophagy-related factor involved in the lipidation of the microtubule-associated protein light chain 3 (LC3)—an essential step for autophagosome formation, expansion, and fusion with lysosomes (Fig. 3B). It also colocalized with LC3 itself, along with the autophagy adaptor protein p62/SQSTM1 (Fig. 3B) (1, 42–45). Lastly, we found that Sp100, a substrate of the ICP0 E3 ubiquitin ligase, was present in the ICP0 structures (Fig. 3B). Sp100 isoforms have been implicated in epigenetic modulation of chromatin, and at least one of these isoforms is upregulated by interferons (IFNs) (2, 13, 46–49). The unsumoylated Sp100A isoform is found in the cytoplasm and may be the one colocalizing with CIN85 (2, 10, 13, 46–49). In similar transfection/infection assays, we found that factors colocalizing with ICP0 also colocalized with CIN85 (Fig. 3C). We also performed a series of control assays to determine whether CIN85 colocalizes with the targets discussed above in uninfected cells (Fig. S2). In uninfected cells, CIN85 formed vesicular structures that were associated with tethering and structural components such as Rab5, Rab7, and LC3. However, the CIN85 structures did not colocalize with the other autophagy components ATG5 and p62/SQSTM1, nor with Sp100A (Fig. S2). This indicated that the observed colocalization events occur specifically during HSV-1 infection. In further support of this specificity, the analysis of additional vesicular markers revealed that neither ICP0 nor CIN85 colocalized with Rab31, which is involved in ligand-bound EGFR trafficking from early to late endosomes (Fig. S3) (50). This is perhaps because Rab5 appears to play a major role in this process, as Rab31 is not the only Rab GTPase involved in EGFR endocytosis (51). Furthermore, ICP0/CIN85 did not colocalize with Rab33b (Fig. S3). This is a Golgi-resident small GTPase that plays a role in the initial stage of autophagy as it is recruited to the isolation membrane through its interaction with ATG16L, a factor which facilitates autophagophore expansion (52). Rab33b is expected to dissociate from the autophagosome, along with ATG16L, prior to its completion which may explain its absence from the ICP0/CIN85 structures. However, we found that approximately 70% of the ICP0/CIN85 vesicles colocalize with Rab27b and 20% with Rab27a (Fig. S4). Both GTPases mediate MVBs docking at the plasma membrane and may facilitate CIN85 cargo exocytosis (53). Notably, Rab27a, Rab27b, Rab31, and Rab33b displayed a more dispersed localization in HSV-1(F)-infected cells compared to uninfected cells, indicating that their function may be altered in HSV-1 infected cells (Fig. S3 and S4).

Furthermore, we did not notice any colocalization of ICP0/CIN85 with DAPRed (Fig. S3B). This is a small fluorescent molecule that specifically accumulates in autophagosomes and autolysosomes, but is not present in early or late endosomes. These findings indicate that the ICP0/CIN85 structures do not display features of autophagosomes.

Finally, we did not notice any colocalization of ICP0/CIN85 with the CD63 tetraspanin (Fig. 3B and C; Fig. S2). Our previous studies determined CD63 as a marker for the major population of EVs released by HSV-1-infected cells (29, 30). These EVs lack viral factors and components of the ESCRT machinery but contain host factors, including STING (31). The absence of colocalization suggests that CIN85 and CD63 belong to different EV populations.

The extent of colocalization of ICP0 and CIN85 with early and late endosomal markers, autophagy-related factors, other vesicular markers, and innate immune components, was evaluated using the Pearson’s correlation coefficient (Fig. S4 and S5). Values closer to 1 indicate stronger colocalization, while values smaller than 0.5 indicate weaker colocalization. Notably, ICP0 and CIN85 colocalize on the surface of the vesicular structures, and consequently, intraluminal cargo displays a low Pearson’s correlation coefficient even though it may be packaged into the vesicles due to ICP0/CIN85. Thus, colocalization with ICP0/CIN85 is not an indicator of whether cargo can be packaged into ICP0/CIN85 structures. Overall, major host defense factors from autophagy, epigenetic silencing, and innate immunity pathways are present in the ICP0/CIN85 structures, highlighting these key viral-host interactions during infection.

### CIN85 is exocytosed during HSV-1 infection, in an ICP0-dependent manner, along with cargo localizing to the CIN85 structures

**Fig 4:**
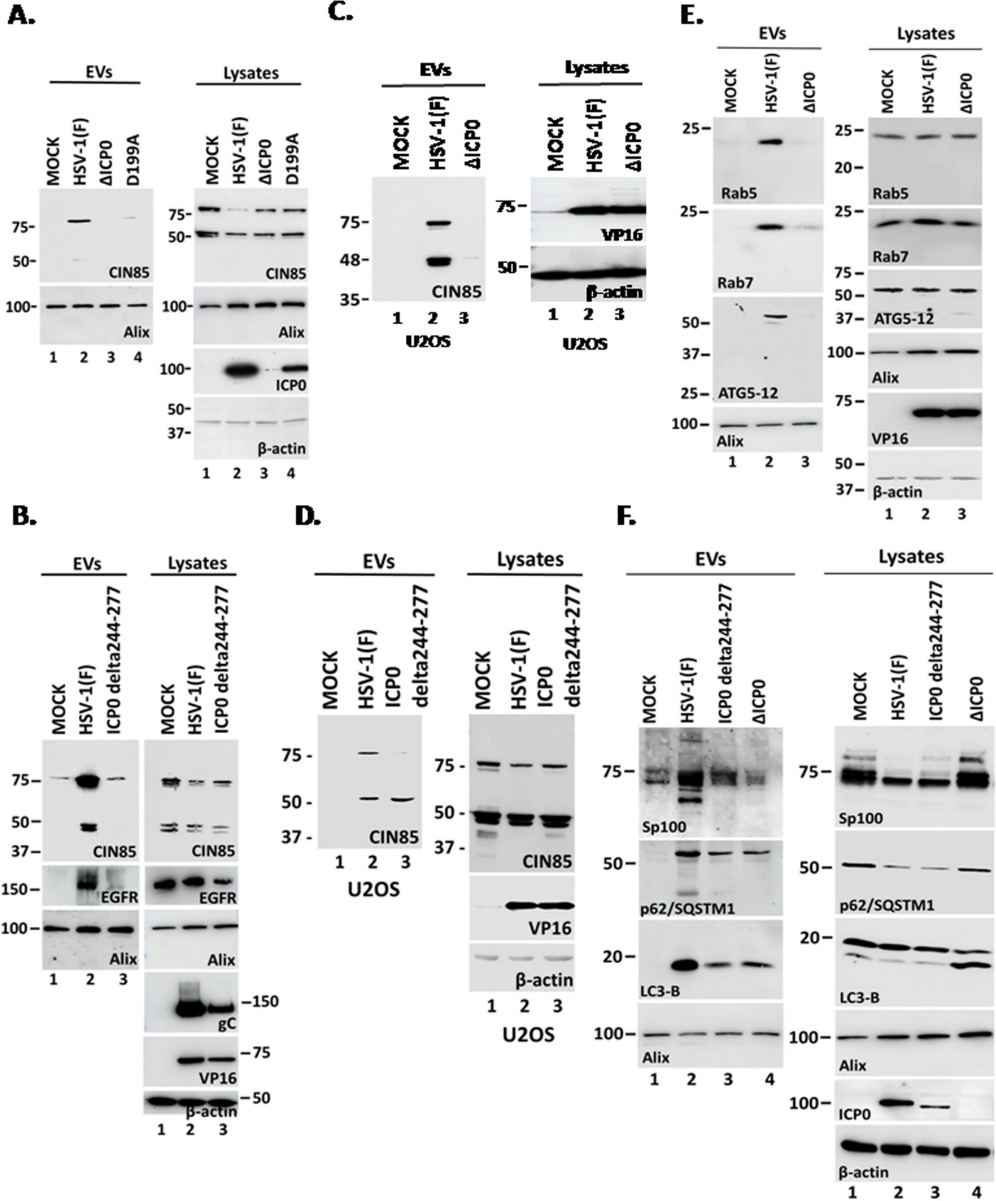
HSV-1(F) infection induces exocytosis of CIN85 and of components colocalizing with CIN85. (**A and Β**) hTERT-HEL cells were uninfected, infected with the WT virus or various ICP0 mutants (0.5 PFU/cell). The cells were harvested at 48 h post-infection, and total EVs were analyzed for the indicated proteins. Equal amounts of proteins from total cell lysates were included as controls. (**C and D**) U2OS cells were infected with HSV-1(F), ΔICP0, and ICP0 delta244–277 virus (0.5 PFU/cell) or remained uninfected. EVs were collected at 48 h post-infection, and equal amounts were analyzed for CIN85 exocytosis. Equal amounts of proteins from total cell lysates were analyzed for CIN85, VP16 which served as a control for the infection, and β-actin which served as a loading control. (**Ε and F**) hTERT-HEL cells were infected with HSV-1(F), ΔICP0, and ICP0 delta244–277 virus (0.5 PFU/cell) or remained uninfected. EVs were collected from culture supernatants at 48 h post-infection and analyzed for Rab5, Rab7, ATG5, Sp100, p62/SQSTM1, Alix, and LC3-B. Equal amounts of proteins from total cell lysates were analyzed for the same proteins. ICP0 or VP16 was used as controls for the infection and β-actin as a loading control.

CIN85 is an endocytosis adaptor, and given the crosstalk between endocytosis and exocytosis, we investigated whether CIN85 participates in an exocytosis pathway in HSV-1-infected cells. hTERT-HEL cells were infected with the WT virus, an ICP0-null mutant, or the D199A mutant virus, where ICP0 remains in the nucleus. We then analyzed EVs for the presence of CIN85. As shown in Fig. 4A, CIN85 was exocytosed from WT virus-infected cells but not from those infected with the two ICP0 mutants, indicating that cytoplasmic ICP0 is required for CIN85 exocytosis. Alix exocytosis is comparable between infected and uninfected cells and has been used as an EV loading control. To determine if the two CIN85-binding motifs of ICP0 are required for CIN85 exocytosis, we repeated the infections with the WT virus and the ICP0 delta244–277 virus. CIN85 exocytosis levels in cells infected with the mutant virus were comparable to those in uninfected cells and significantly lower than in WT-infected cells, indicating that the two CIN85-binding motifs of ICP0 are required for CIN85 exocytosis (Fig. 4B). We also assessed the exocytosis of EGFR, a receptor that was found to be internalized during HSV-1 infection in an ICP0-dependent but ligand-independent manner (22). EGFR exocytosis occurred in WT virus-infected cells but was absent in those infected with the ICP0 delta244–277 virus (Fig. 4B). Again, Alix was used as an EV loading control. To rule out the possibility that the lack of CIN85 exocytosis resulted from growth defects of the ICP0 mutant viruses in immunocompetent cells, we repeated these assays in U2OS cells that are known to support the growth of ICP0 mutants (54, 55). As shown in Fig. 4C, ΔICP0 did not promote CIN85 exocytosis in U2OS cells, unlike WT virus. Interestingly, in ICP0 delta244–277-infected U2OS cells, the longer CIN85 isoform was not exocytosed, but a shorter variant was detected in EVs (Fig. 4D). We noticed that this shorter variant is the dominant form of CIN85 in U2OS cells (Fig. 4D). Based on antibody analysis, this CIN85 variant originates from the C-terminus of the protein and, therefore, is still able to anchor to the membranes but appears to lack SH3 domains that are critical for the endocytosis and cargo sorting functions of CIN85. U2OS are cancer cells, derived from a human osteosarcoma, and an enrichment in this sorter CIN85 variant is expected to interfere with rapid surface receptor endocytosis, allowing for prolonged signaling. Notably, some receptors turned off via the CIN85 pathway, including EGFR, FGFR, and RTKs, are important for tumor growth. Overall, these data indicate that the binding of ICP0 to CIN85 is integral to CIN85 and EGFR exocytosis during the course of HSV-1 infection.

Given that CIN85 was exocytosed during HSV-1(F) infection, we investigated whether other proteins localizing to the CIN85 structures were also present in EVs. hTERT-HEL cells were infected with WT, ICP0 delta244–277, or ΔICP0 viruses, and EVs were collected from culture supernatant and analyzed for the proteins found in the CIN85 structures from Fig. 3. We found that Rab5 (early endosomal marker), Rab7 (late endosomal marker), along with the ATG5–ATG12 conjugate complex (functions as an E3-like enzyme to promote LC3 lipidation), were exocytosed in WT virus-infected cells but not in ΔICP0-infected cells (Fig. 4E). Alix exocytosis was indistinguishable under these conditions. We previously reported that the autophagy adaptor protein p62/SQSTM1, which directs substrates to autophagosomes, was degraded as early as 3 h post-infection in WT virus-infected cells, through an ICP0-dependent mechanism (56). In addition, a fraction of p62/SQSTM1 was detected in EVs released by WT virus-infected cells (Fig. 4F). This p62/SQSTM1 degradation along with its exocytosis likely represents a complementary mechanism of WT virus to effectively block autophagy. In contrast, the ΔICP0 virus cannot effectively block autophagy, leading to p62/SQSTM1 degradation through the autophagy pathway, with only a small fraction being exocytosed. While the ICP0 delta244–277 virus retains the ability to degrade p62/SQSTM1 like the WT virus, it displays reduced p62/SQSTM1 exocytosis, likely due to the disruption of CIN85-mediated exocytosis. Furthermore, in ICP0 delta244–277 virus-infected cells, low levels of autophagy may contribute to additional decrease in p62/SQSTM1 (Fig. 4F). Supporting these findings, we observed an increased LC3-II/I lipidation ratio in the lysates from ICP0-null virus-infected cells, indicating ongoing autophagy (Fig. 4F). This likely explains the reduced LC3-II exocytosis in ΔICP0-infected cells. In contrast, WT virus efficiently counteracts autophagy, leading to low intracellular LC3-II levels and higher exosomal LC3-II levels (Fig. 4F). ICP0 delta244–277 virus-infected cells frequently exhibited intermediate LC3 lipidation, indicating low autophagy levels coupled with an inability to cause LC3-II exocytosis.

We also monitored Sp100 exocytosis, a component of ND10 bodies that is involved in epigenetic silencing of viral chromatin. Both WT and ICP0 delta244–277 viruses degraded selected Sp100 forms (Fig. 4F; Fig. S1), but only WT virus induced exocytosis of Sp100. Neither exocytosis nor degradation of Sp100 was observed in ΔICP0-infected cells (Fig. 4F). Overall, these findings indicate that cargo from CIN85 structures could be exocytosed in an ICP0-dependent manner.

### CIN85 is required for increased CD63 exocytosis during HSV-1(F) infection, while ATG5 is required for CIN85 exocytosis

**Fig 5:**
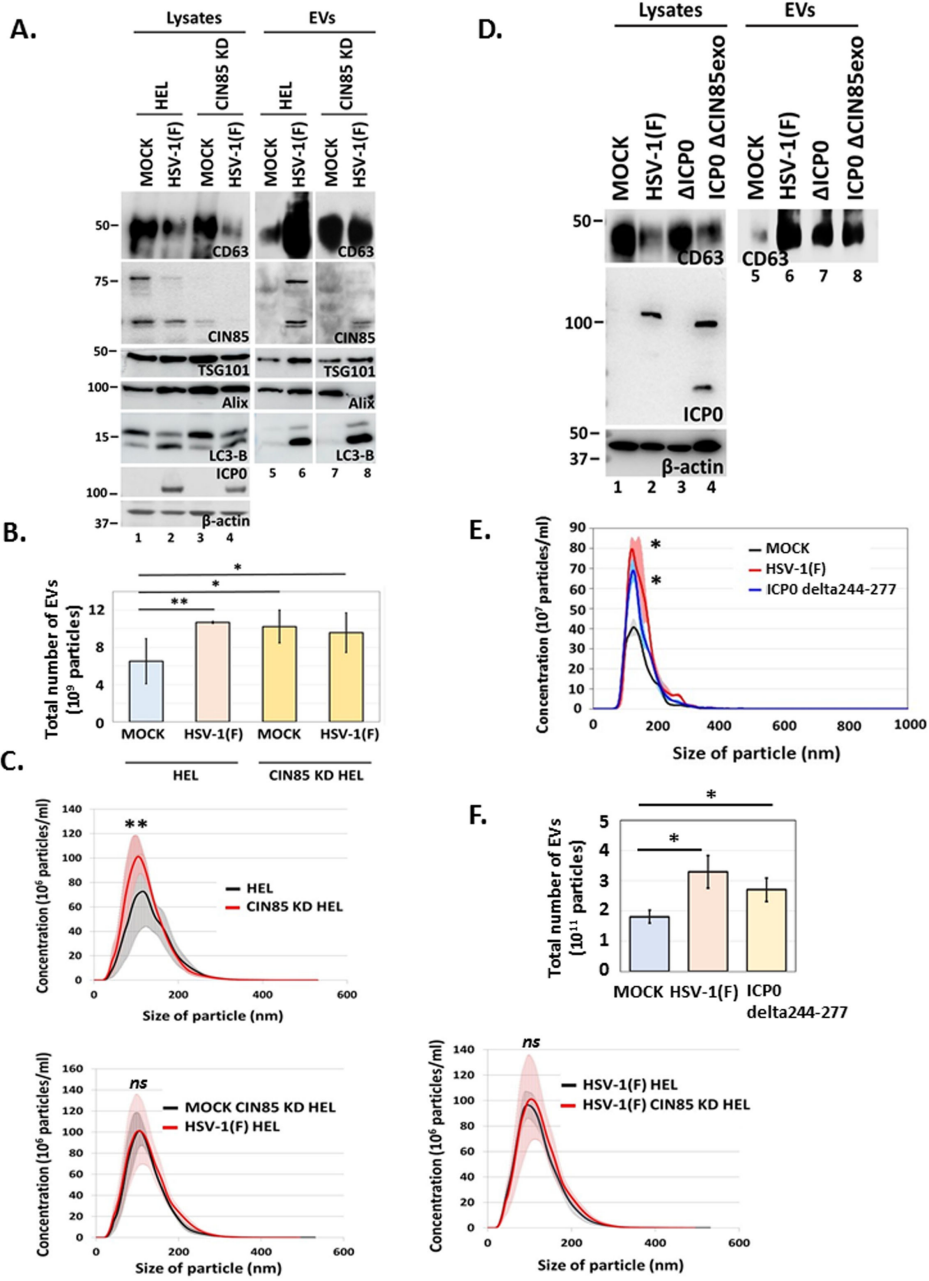
CIN85 is required for CD63 exocytosis during HSV-1 infection. (**A**) HEL cells and the CIN85 KD derivatives were infected with HSV-1(F) (0.5 PFU/cell). EVs and equal amounts of cell lysates were analyzed at 48 h post-infection for CD63, CIN85, TSG101, ALIX, and LC3-B. ICP0 was used as a control for the infection and β-actin as a loading control. (**B and C**) HEL cells and the CIN85 KD derivatives were infected with the WT virus (0.5 PFU/cell) or remained uninfected. EVs were isolated at 48 h post-infection through an iodixanol/sucrose gradient, described in Materials and Methods, and quantified by NTA. Total number of EVs (**B**) and size distribution (**C**) are depicted. (**D**) HEL cells were infected with HSV-1(F), ΔICP0, ICP0 delta244–277 virus (0.5 PFU/cell), or remained uninfected. At 48 h post-infection, EVs and cell lysates were analyzed for CD63. ICP0 served as a control for the infection and β-actin as a loading control. (**E and F**) HEL cells were infected with HSV-1(F), ICP0 delta244–277 virus (0.5 PFU/cell) or remained uninfected. EVs were isolated at 48 h post-infection as described in panel A and quantified by NTA. The size distribution (**E**) and total number of EVs (**F**) are depicted. *P < 0.05,**P < 0.01, ***P < 0.001, ****P < 0.0001.

We have previously demonstrated that WT virus infection triggers increased production of EVs through the CD63 biogenesis pathway (29, 30, 57). Since CIN85 does not colocalize with CD63 (Fig. 3; Fig. S2), we sought to determine whether it has a role in CD63 exocytosis. For this, we developed a CIN85 KD HEL cell line and compared CD63 exocytosis between parental and CIN85 KD cells following HSV-1(F) infection. We found that uninfected CIN85 KD cells displayed higher basal levels of exosomal CD63 than parental cells (Fig. 5A). However, WT virus infection failed to further induce CD63 exocytosis in CIN85 KD cells compared to parental cells (Fig. 5A). We also quantified the total EVs released from hTERT-HEL cells and compared them to CIN85 KD derivatives. Consistent with our previous findings, we confirmed that in the parental cells HSV-1 infection stimulated almost a twofold higher production of EVs compared with uninfected cells (Fig. 5B). CIN85 KD cells produced almost twice the number of EVs compared with the parental cells at baseline, but WT virus did not stimulate further EV production (Fig. 5B and C). The size distribution of EVs was comparable between infected and uninfected hTERT-HEL and CIN85 KD derivatives. These results support that CIN85 regulates CD63+EV biogenesis during WT virus infection. To further investigate EV cargo differences, a targeted approach was used to compare EV cargo from infected and uninfected hTERT-HEL cells with those from CIN85 KD derivatives. Beyond the previously discussed differences in CD63 exocytosis, we noticed that ALIX exocytosis was reduced in infected CIN85 KD cells, whereas TSG101 exocytosis remained unchanged (Fig. 5A). ALIX is a scaffold protein of ESCRT-II that facilitates cargo sorting and recruits the ESCRT-III machinery for vesicle formation, while TSG101, an ESCRT-I component, is involved in cargo recognition during the early stages of intraluminal vesicle formation. This suggests CIN85 selectively regulates ESCRT components like ALIX. Supporting this, previous studies have identified CIN85 as an ALIX interactor with shared binding partners (58, 59). Notably, exocytosis of the autophagosome component, LC3-B, was unaffected between infected hTERT-HEL and CIN85 KD cells, indicating its independence from CIN85 (Fig. 5A).

In a reciprocal approach, we tested whether the ICP0 mutants, ΔICP0 and ICP0 delta244–277, could induce CD63 exocytosis like WT virus. Infections were performed in hTERT-HEL cells, EVs were isolated from culture supernatants and analyzed for CD63. Our findings revealed that neither ICP0 delta244–277 mutant nor ΔICP0 virus could induce CD63 exocytosis to WT virus levels (Fig. 5D). Particularly, ΔICP0 virus has reduced ability to counteract autophagy (Fig. 4F). Instead of diverting CD63+ endosomes for exocytosis, as the WT virus does, ΔICP0 redirects them to autophagosomes for degradation (30, 33). These results suggest that CIN85 plays a role in increased production of CD63+EVs during HSV-1 infection.

**Fig 6:**
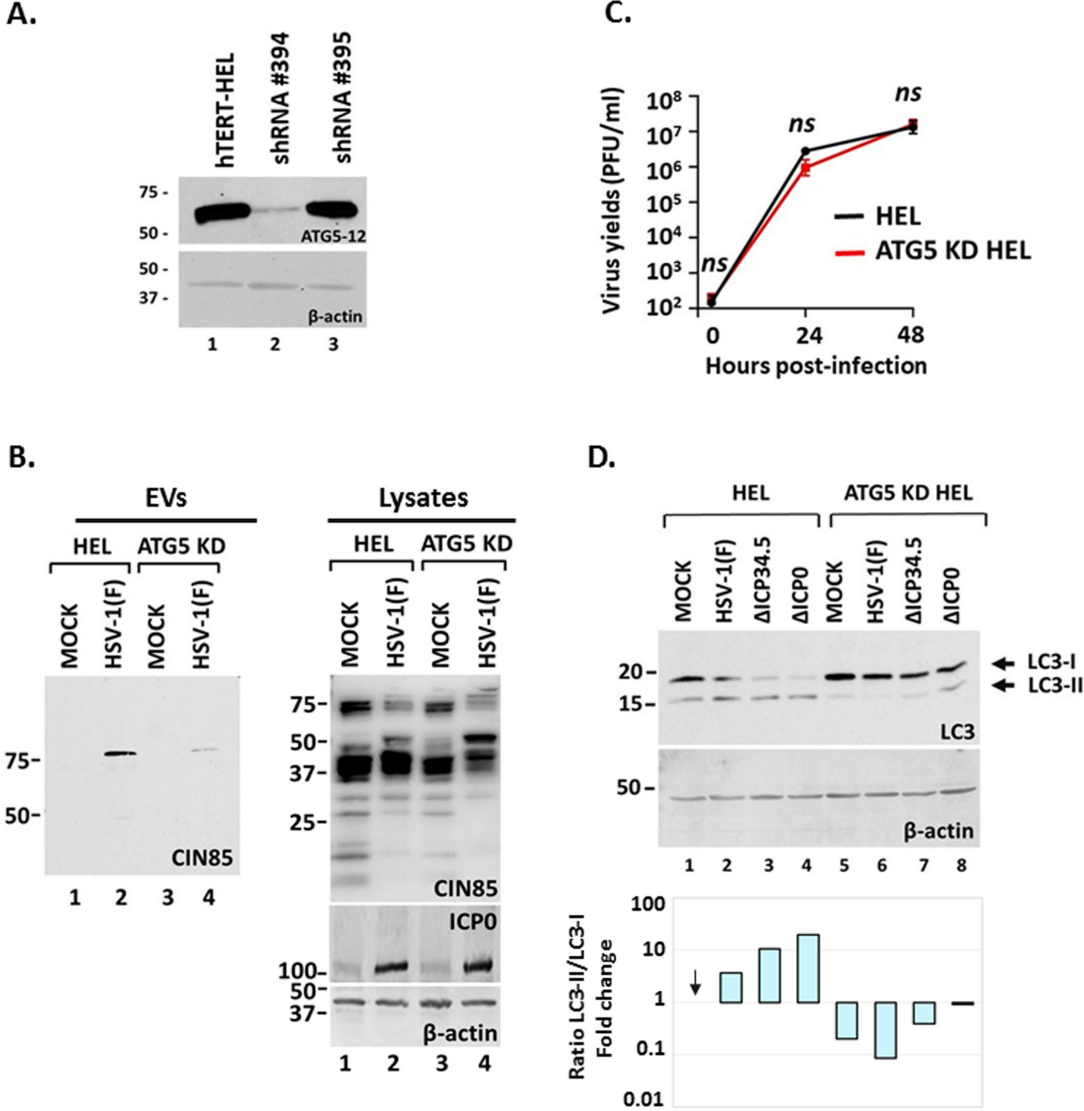
ATG5 is required for CIN85 exocytosis during HSV-1 infection. (**A**) Efficiency of ATG5 depletion by shRNA #394 is depicted. Protein analysis was done as above. (**B**) HEL cells and the ATG5 KD derivatives were infected with HSV-1(F) (0.5 PFU/cell) or remained uninfected. At 48 h post-infection, EVs and equal amounts of cell lysates were analyzed for CIN85. ICP0 served as a control for the infection and β-actin as a loading control. (**C**) hTERT-HEL cells and the ATG5 KD derivatives were infected with the WT virus (0.01 PFU/cell). The cells were harvested at 3, 24, and 48 h post-infection, and progeny virus production was quantified by plaque assays. (**D**) hTERT-HEL cells and the ATG5 KD derivatives were infected with HSV-1(F), ΔICP34.5, and ΔICP0 virus (0.5 PFU/cell) or remained uninfected. The cells were harvested at 24 h post-infection, and equal amounts of proteins from total cell lysates were analyzed for LC3 lipidation, whereas β-actin served as a loading control. The LC3-II and LC3-I signals were quantified using ImageJ and normalized to β-actin. The ratio of LC3-II to LC3-I for each sample relative to the ratio in uninfected hTERT-HEL cells is depicted.

Finally, we investigated the role of autophagy in CIN85 exocytosis during HSV-1 infection since mutant viruses that failed to evade autophagy (such as ΔΙCP0) also failed to induce CIN85 exocytosis (Fig. 4). To test this, we developed an hTERT-HEL cell line depleted of ATG5. The efficiency of ATG5 depletion is depicted in Fig. 6A. We discovered that ATG5 depletion suppressed CIN85 exocytosis during HSV-1 infection (Fig. 6B), but this was not due to defects in viral replication, as virus growth was unaffected in ATG5 KD cells (Fig. 6C). The ratio of LC3-II/LC3-I lipidation, a key measure of autophagy, was not increased by defective viruses in ATG5 KD cells compared to the parental cells, showing that autophagy was effectively blocked in these cells (Fig. 6D). Overall, we uncovered a crosstalk between endocytosis, autophagy, and exocytosis during HSV-1 infection, where CIN85 is required for increased CD63 exocytosis by WT virus, while ATG5 regulates CIN85 exocytosis.

### CIN85 is required for suppression of antiviral responses during HSV-1 infection and for optimal virus yields

**Fig 7:**
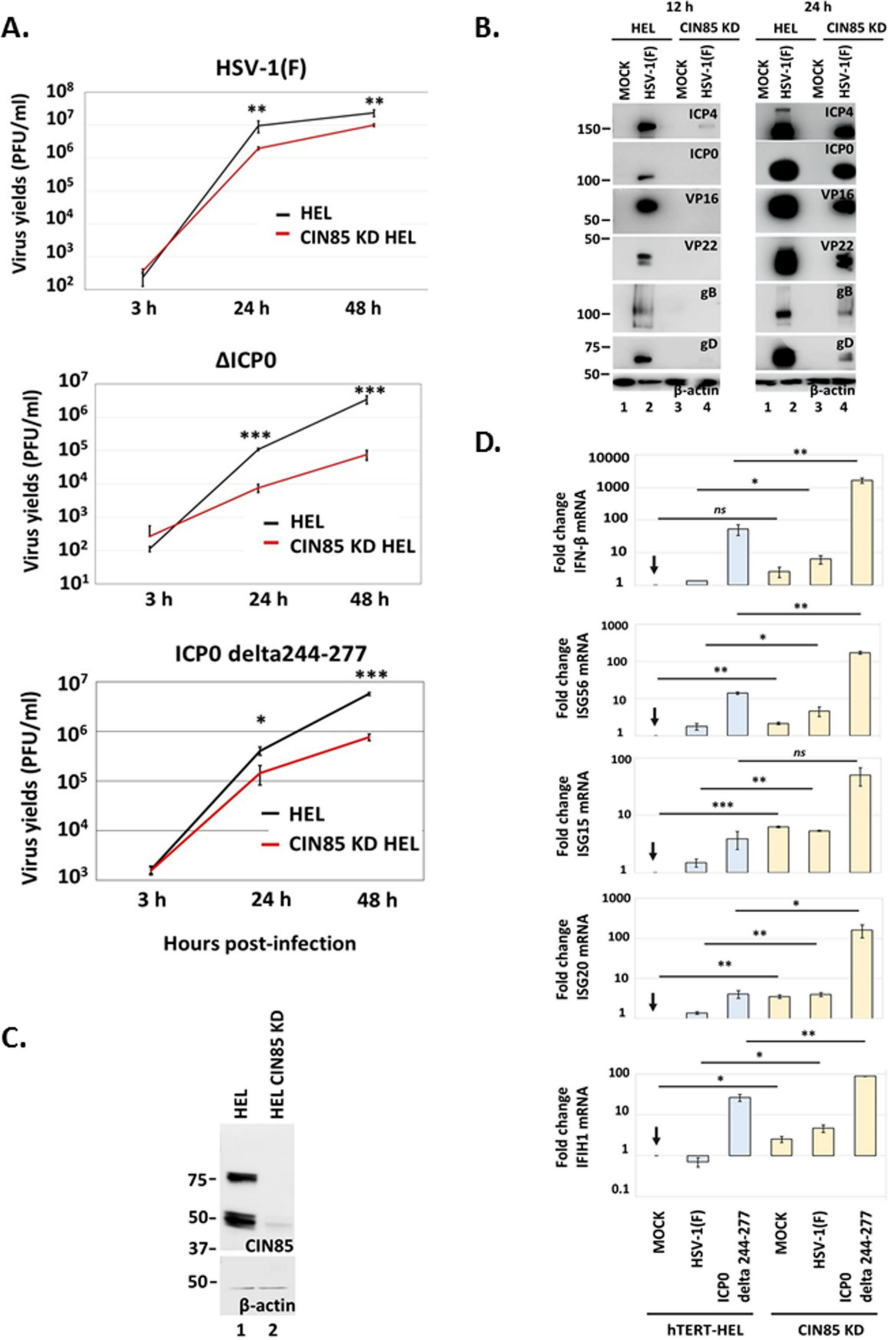
Growth defects of WT virus in CIN85 KD HEL cells. (**A**) hTERT-HEL cells and the CIN85 KD derivatives were infected with the WT virus, a ΔICP0 virus, or the ICP0 delta244–277 virus (0.01 PFU/cell). The cells were harvested at 3, 24, and 48 h post-infection, and progeny virus production was quantified in Vero cells for the WT virus, and in U2OS cells for the ICP0 mutants. (**B**) hTERT-HEL cells were infected with HSV-1(F) (0.5 PFU/cell) or remained uninfected. The cells were harvested at 12 and 24 h post-infection, and equal amounts of proteins from total cell lysates were analyzed for ICP4, ICP0, VP16, VP22, gB, gD, and β-actin that served as a loading control. (**C**) Efficiency of CIN85 depletion in hTERT-HEL cells treated with a CIN85 shRNA. Equal amounts of proteins from total cell lysates were analyzed for CIN85, whereas β-actin served as a loading control. (**D**) hTERT-HEL cells and the CIN85 KD derivatives were infected with HSV-1(F), the ICP0 delta244-277 virus (0.1 PFU/cell) or remained uninfected. The cells were harvested at 8 h post-infection, total RNA was extracted, and innate immunity and pro-inflammatory gene expression was assessed by RT-qPCR. *P < 0.05, **P < 0.01, ***P < 0.001, ****P < 0.0001.

Since deletion of the two CIN85-binding motifs of ICP0 reduced progeny virus production, we further assessed viral yields in CIN85 KD cells. hTERT-HEL cells and CIN85 KD derivatives were infected with HSV-1(F) (0.01 PFU/cell), and viral progeny was quantified at 3, 24, and 48 h post-infection by plaque assays. We observed an approximately eightfold decrease in WT virus yields in CIN85 KD cells compared to parental cells, indicating that CIN85 has a proviral role (Fig. 7A). In addition, we assessed progeny virus production in hTERT-HEL and CIN85 KD cells infected with ΔICP0 and ICP0 delta244–277 viruses. Since the WT virus employs multiple mechanisms to counteract antiviral responses, which could mask the importance of CIN85 depletion, we chose to simultaneously deplete CIN85 and its interactor, ICP0. Infections were performed at low multiplicity, and samples were harvested at 3, 24, and 48 h post-infection to quantify progeny virus production. Both viruses displayed a 15- to 20-fold decrease in progeny virus production in CIN85 KD HEL compared to parental cells (Fig. 7A). Additionally, we assessed the impact of CIN85 depletion on HSV-1(F) gene expression. hTERT-HEL and CIN85 KD cells were infected with HSV-1(F) (0.5 PFU/cell) or remained uninfected. Replicate cultures were harvested at 12 and 24 h post-infection, and equal amounts of proteins from total cell lysates were analyzed for ICP4, ICP0, VP16, VP22, gB, and gD. CIN85 KD cells exhibited a substantial delay in viral gene expression compared to parental cells (Fig. 7B), further supporting CIN85’s role in optimal viral gene expression. The efficiency of CIN85 depletion is depicted in Fig. 7C.

To determine the mechanism of virus restriction in CIN85-depleted cells, we monitored activation of innate immunity gene expression. The hTERT-HEL cells and the CIN85 KD derivatives were infected with HSV-1(F), the ICP0 delta244–277 virus (0.1 PFU/cell), or remained uninfected. The cells were harvested at 8 h post-infection and analyzed for innate immunity gene expression. As shown in Fig. 7D, basal levels of IFN-β and interferon-stimulated genes (ISGs) were higher in CIN85 KD cells compared to the parental cells. Furthermore, both wild type and ICP0 delta244–277 virus displayed higher innate immunity gene expression in CIN85 KD cells compared to hTERT-HEL cells (Fig. 7D).

**Fig 8:**
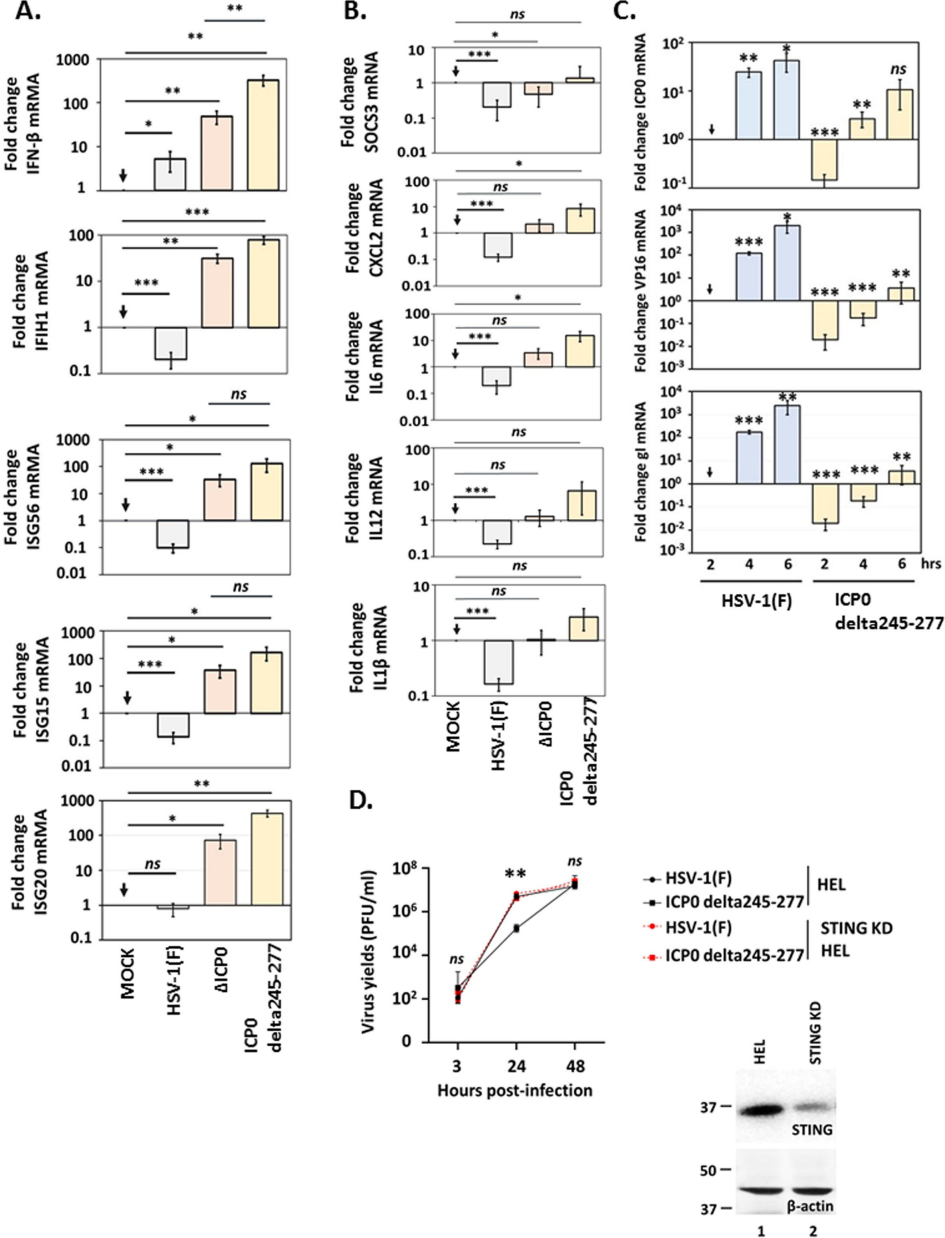
Activation of innate immunity and inflammatory responses in cells infected with ICP0 delta244–277 virus. (**A and B**) hTERT-HEL cells were infected with the WT virus, ΔICP0, ΙCP0 delta244–277 (0.3 PFU/cell) or remained uninfected. The cells were harvested at 14 h post-infection, total RNA was extracted, and innate immunity (**A**) and pro-inflammatory gene expression (**B**) was assessed by RT-qPCR. (**C**) hTERT-HEL cells were infected with HSV-1(F) or ICP0 delta244–277 virus (0.1 PFU/cell). The cells were harvested at 2, 4, and 6 h post-infection, total RNA was extracted, and quantification of viral gene expression (ICP0, VP16, and gI) was done by RT-qPCR. (**D**) hTERT-HEL cells and STING KD derivatives were infected with HSV-1(F) or ΙCP0 delta244–277 virus (0.01 PFU/cell). The cells were harvested at 3, 24, and 48 h post-infection, and progeny virus production was quantified in U2OS cells. The efficiency of STING depletion in HEL cells was assessed by western blot. For all assays, three biological replicates were analyzed. Statistical significance was evaluated using student’s *t*-test as described in Materials and Methods. *P < 0.05, **P < 0.01, ***P < 0.001, ****P < 0.0001.

In a reciprocal approach, to determine the mechanism of ICP0 delta244–277 restriction, we monitored the activation of innate immunity gene expression, which is an indication of the ability of the virus to evade host antiviral responses. We performed these studies in immortalized hTERT-HEL cells since they have intact innate immunity pathways, maintain contact inhibition, and can undergo senescence. Replicate cultures of hTERT-HEL cells were infected with HSV-1(F) or ICP0 delta244–277 at a low multiplicity of infection, wherein the virus is predominately utilizing viral gene expression for immune evasion vs protein delivery within the virions. Uninfected cells served as a negative control, while ΔICP0-infected cells (that cannot counteract antiviral responses) were used as a positive control. Samples were harvested at 14 h post-infection for quantification of innate immunity genes. RT-qPCR analysis revealed that ICP0 delta244–277 infection led to significant induction of IFN-β along with IFN-stimulated genes, including the interferon-induced helicase-1 (IFIH1), ISG56, ISG15, and ISG20 (Fig. 8A). This response was comparable between ICP0 delta244–277 and ΔICP0 virus-infected cells, indicating that ICP0 amino acids 244–277 are crucial for its immunoevasion function. Conversely, inflammatory genes including the suppressor of cytokine signaling-3 (SOCS3), the C-X-C motif chemokine ligand 2 (CXCL2), along with the interleukins IL6, IL12, and IL-1β, were only mildly induced by ICP0 mutant viruses compared to uninfected cells (Fig. 8B). Unlike mutant viruses, the WT virus effectively counteracted antiviral responses, reducing type I IFN and inflammatory genes expression below basal levels found in uninfected cells (Fig. 8A and B).

Given that ICP0 delta244–277 virus-infected cells activate innate immune pathways and ICP0 delta244–277 virus displays growth defects at low multiplicity of infection, we analyzed ICP0 delta244–277 gene transcription relative to WT virus. hTERT-HEL cells were infected with the WT virus or ICP0 delta244–277 (0.1 PFU/cell). Total RNA was extracted at 2, 4, and 6 h post-infection, and quantification of viral transcripts such as ICP0 (α-gene), VP16 (γ-gene), and gI (γ-gene) was performed. RT-qPCR analysis revealed that ICP0 transcripts were approximately 10-fold lower in ICP0 delta244–277 compared with the WT virus-infected cells, while late gene transcripts were 100- to 1,000-fold less. These findings underscore the importance of the ICP0 amino acids 244–277 for optimal viral gene expression.

To ensure that the growth defect of ICP0 delta244–277 virus at low multiplicity of infection was due to innate immunity activation and not due to other mechanisms, we tested whether this effect could be rescued in immunodeficient STING KD cells. STING (stimulator of interferon genes) is a DNA sensor that, upon activation, triggers type I IFN and pro-inflammatory responses, acting as a major restriction factor against HSV-1 and other pathogens. hTERT-HEL cells and STING KD cells were infected with HSV-1(F) or ICP0 delta244–277 virus (0.01 PFU/cell). The cells were harvested at 3, 24, and 48 h post-infection, and progeny virus production was quantified by plaque assays in U2OS cells. As shown in Fig. 8D, the growth defect of ICP0 delta244–277 virus could be rescued in STING KD cells, further indicating that innate immunity activation is the primary mechanism restricting ICP0 delta244–277 virus. The efficiency of STING depletion is also depicted.

## DISCUSSION

ICP0 has been studied for more than six decades, with an emphasis on its nuclear functions and its role in activating viral gene transcription by degrading organizers of the ND10 bodies, dispersing repressor complexes, recruiting chromatin remodeling enzymes, and suppressing other innate immunity factors (2–4, 60–63). However, the significance of its cytoplasmic accumulation following virus replication, and the nature of the punctate structures where it accumulates, has remained an enigma. The discovery of the ICP0/CIN85 interaction and ICP0-dependent internalization of EGFR in a ligand-independent manner have indicated that ICP0 may be involved in endocytosis processes (22). Further supporting this, we previously determined that the virus entry receptor, nectin-1, is internalized during HSV-1 infection via the same complex to facilitate virus spread (28). These observations were instrumental and prompted us to further explore the role of ICP0 in cargo trafficking through various membrane compartments, including endosomes, autophagosomes, and extracellular vesicles.

The salient findings of our studies include the localization of ICP0 relative to CIN85, the nature of the CIN85 structures, and the importance of the ICP0/CIN85 interaction in HSV-1 infection. ICP0 was found on the surface of vesicular structures where CIN85 localizes. CIN85 docks on endosomal membranes via its positively charged C-terminus, which interacts with anionic phospholipids on the cytoplasmic side of the membrane, and via its coiled-coil domain, which binds phosphatidic acid (64). ICP0 likely binds to CIN85 on the surface of these vesicles. During HSV-1 infection, CIN85 vesicles were found to contain small GTPases, such as Rab5, which regulates early endosomal trafficking, and Rab7, which controls early to late endosome and autophagosome maturation, fusion of late endosomes to the trans-Golgi network, as well as fusion of autophagosomes with lysosomes. Also, some ICP0/CIN85 vesicles colocalized with the two Rab27 isoforms (Rab27a and Rab27b) involved in MVB docking to the plasma membrane, and this is consistent with their exocytosis (53). However, several other small GTPases showed no association with the ICP0/CIN85 structures. For instance, Rab31 did not colocalize with ICP0/CIN85. This small GTPase controls a non-canonical, ESCRT-independent pathway, which sorts EGFR into MVBs and subsequently to EVs. This pathway of EGFR exocytosis antagonizes the canonical pathway of EGFR trafficking, where the internalized receptor is ubiquitinated by the ESCRT machinery, sorted to MVBs, and then fused with lysosomes resulting in EGFR degradation (50). Since Rab31 was found not to colocalize with ICP0/CIN85, it suggests perhaps a different mechanism controls EGFR exocytosis during HSV-1 infection. Alternatively, a small amount of Rab31, potentially associated with the ICP0/CIN85 structures, may be sufficient for EGFR exocytosis. Additionally, we investigated Rab33b for potential colocalization with the ICP0/CIN85 structures. We used the Rab33b ortholog from zebrafish (Rab33bb), which has almost 70% identity to the human ortholog, as it was immediately available in our lab. Human Rab33b has a role in the early stages of autophagy by recruiting the ATG12-ATG5-ATG16L1 complex to phagophores to promote their expansion (52). No colocalization was observed between ICP0 and Rab33bb. Given that Rab33b and ATG16L1 dissociate from autophagosomes upon their maturation, the absence of CIN85/Rab33bb colocalization was expected, even if CIN85 vesicles were related to autophagosomes (52). Unlike these small GTPases, we discovered that ICP0/CIN85 structures colocalize with markers of autophagosomes, including ATG5, LC3, and the autophagy adaptor protein p62/SQSTM1. LC3 was present in the CIN85 vesicles in both infected and uninfected cells, whereas ATG5 and p62/SQSTM1 were recruited only in infected cells. WT virus has evolved mechanisms to block autophagy, as indicated by low levels of LC3 lipidation compared with uninfected cells, and the degradation of several autophagy adaptors during the early stages of HSV-1 infection. This is further supported by the lack of staining of the CIN85 structures with the autophagic vacuole dye DAPRed. DAPRed is a small fluorescent molecule used to detect autophagosomes and autolysosomes by incorporating into the autophagic vesicles during double membrane formation and then emitting fluorescence under hydrophobic conditions. Together, these findings support that CIN85 structures are likely not autophagosomes (65–72). Finally, we discovered that the factors localizing in the CIN85 structures were also present in EVs released by infected cells, suggesting that the CIN85 vesicles facilitate cargo exocytosis during HSV-1 infection.

Another seminal aspect of our studies concerns the crosstalk between endocytosis, autophagy, and exocytosis in HSV-1-infected cells. We have extensively published that HSV-1 infection induces EV biogenesis through the CD63 pathway, but CIN85 does not colocalize with CD63 indicating that they belong to different vesicle populations (29, 30). However, we discovered that CIN85 has a regulatory role on the CD63 EV biogenesis pathway. Particularly, CIN85 KD cells displayed higher basal levels of extracellular CD63 compared to uninfected parental cells, whereas HSV-1 infection failed to further stimulate CD63 exocytosis. The ICP0 delta244–277 virus produced less total EVs compared to the WT virus and less CD63+ EVs. Also, this virus failed to promote exocytosis of CIN85 and associated cargo. These data support a crosstalk between the CIN85 endocytosis and the CD63 exocytosis pathways. Additionally, we found that ATG5, an autophagy-related factor, is essential for CIN85 exocytosis during HSV-1 infection (73, 74). ATG5 interacts with the clathrin-associated endocytic machinery and controls endocytic cargo flux, dependent on nutrient availability (73, 74). CIN85 is also implicated in clathrin-coated vesicle formation (75). This perhaps represents the mechanism by which ATG5 regulates CIN85 trafficking. Alternatively, CIN85 retention in the absence of ATG5 may compensate for impaired autophagy by performing sorting functions. Nevertheless, these findings uncovered a novel crosstalk between autophagy and exocytosis during HSV-1 infection.

Finally, our study underscores the importance of ICP0 aa 244–277 and CIN85 during HSV-1 infection. Our hypothesis is that HSV-1 infection via ICP0 and CIN85 redirects selected host defense factors for exocytosis to evade antiviral responses in the cells where it replicates. ICP0, itself, has not been detected in EVs released by infected cells. The ICP0 delta244–277 virus exhibited reduced virus yields, likely due to its inability to suppress IFN-β and interferon-stimulated genes (ISGs), which modestly enhanced autophagy. However, this mutant virus did not stimulate pro-inflammatory responses, unlike IFNs and ISGs, indicating that ICP0 delta244–277 could still evade inflammatory pathways. Consistently, CIN85 KD cells infected with the WT virus activated stronger antiviral responses compared to the parental cells. Among the factors associated with the ICP0/CIN85 structures was Sp100A, a nuclear protein involved in viral gene transcriptional regulation. While ICP0 degrades Sp100 to disperse ND10 bodies, the unsumoylated Sp100A resides in the cytoplasm and is not degraded. Sp100 forms were found in EVs released by HSV-1-infected cells, with its exocytosis occurring in an ICP0-dependent manner. Additionally, several autophagy-related factors found in ICP0/CIN85 structures were also exocytosed. While both ICP0 aa 244–277 and CIN85 were found to have roles in innate immunity suppression, the combination of ICP0 deletion and CIN85 depletion had a greater negative impact on virus growth. This is possible if a CIN85 ortholog, such as CD2AP, partially compensates for CIN85 loss. Also, the higher basal levels of antiviral responses in CIN85 KD cells compared to parental cells could pose a negative effect on an HSV infection that may be more severe for a defective virus. An alternative interpretation is that the proline-rich region of ICP0 between aa 244–277 may have additional roles, or this ICP0 deletion impacts other functions of the protein.

**Fig 9:**
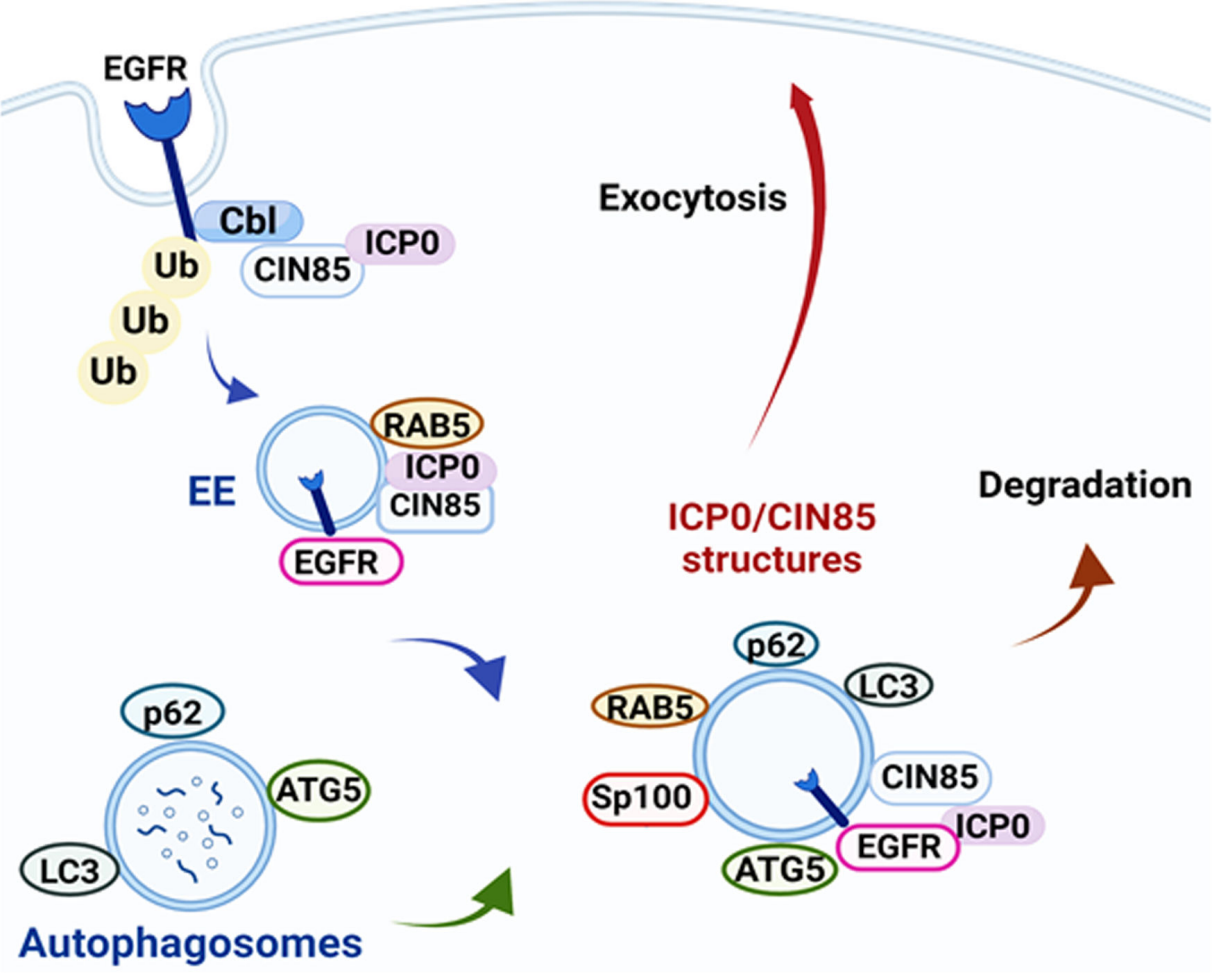
Model for the role of HSV-1 ICP0 in endocytosis, autophagy, and exocytosis. ICP0 forms a complex with CIN85 and Cbl that promotes surface receptor internalization. ICP0 and CIN85 colocalize on the surface of vesicular structures in the cytoplasm of infected cells where autophagy and innate immune components are also recruited. Cargo associated with the CIN85 structures is targeted either for exocytosis through extracellular vesicles or for degradation. This likely represents an immunoevasion strategy of the virus.

Overall, we have identified that the promiscuous transactivator of HSV-1, ICP0, manipulates endosomal, autophagy, and exocytosis pathways to evade host antiviral defenses (see model in Fig. 9). Our findings provide the first evidence that changes in ICP0 localization from the nucleus to the cytoplasm following viral replication have functional significance; further highlighting the pleiotropic roles of this viral protein in different compartments, and altogether in shaping the microenvironment of infection.

## MATERIALS AND METHODS

### Cell lines, viruses, and chemicals

hTERT-HEL cells (immortalized human embryonic lung fibroblasts, human telomerase reverse transcriptase [hTERT] transformed) were cultured in Dulbecco’s modified Eagle’s medium supplemented with 10% fetal bovine serum (FBS) (76). Vero, HEp-2, and HEK-293T cells (ATCC) were cultured according to the manufacturer’s instructions. HSV-1(F) is a limited passage isolate, as described before. The properties of the ΔICP0 (R8501), ΔICP34.5 (R3616), and D199A (R7914) viruses were described before (7, 39, 76, 77). For the development of the ICP0 delta244–277 virus, the ICP0 cDNA with the 244–277 aa deletion was inserted into the PKO5 plasmid (78). A ΔΙCP0 Bac system was used to insert this deletion in the viral genome, as has been described before (7–9).

### Development of stable cell lines and transfection assays

shRNA plasmids for the depletion of human CIN85, STING, and ATG5 were purchased from Sigma. For the development of the respective lentiviral vectors, HEK-293T cells seeded in a F25 cm^2^ flask at a 60% confluency were cotransfected with 8 µg of the plasmid carrying the shRNA, 8 µg of the Gag-Pol-expressing plasmid, and 1 µg of the VSV-G-encoding plasmid with Lipofectamine 3000, according to manufacturer’s instruction (Thermo Fisher Scientific). At 48 h after transfection, the supernatant from the cultures was collected, filtered through 45-μΜ pore-size filters, and used to infect hTERT-HEL cells in the presence of polybrene (8 µg/mL). Puromycin selection (8 µg/mL) was initiated 24 h after exposure to lentiviruses and continued until only resistant clones emerged. The clones of hTERT-HEL cells, with the greater depletion in protein of interest, were used for those studies. The stable lines were maintained in the presence of puromycin, which was removed 24 h before assays were performed.

For the transfection assays, the ratio of plasmid(s) to Lipofectamine 3000 for a given number of cells was calculated according to the manufacturer’s instructions. All plasmids used in this study are listed in Table 1.

**TABLE 1:** List of plasmids used

Plasmids	Vendor	Catalog number	Acknowledgment
mRFP-Rab7	Addgene	#14436	mRFP-Rab7 was a gift from Ari Helenius (79)
pmCherry-ATG5	Addgene	#13095	pmCherry-ATG5 was a gift from Roberta Gottlieb (80)
mCherry-Rab5a-7	Addgene	#55126	mCherry-Rab5a-7 was a gift from Michael Davidson
mCherry-hLC3B-pcDNA3.1	Addgene	#40827	mCherry-hLC3B-pcDNA3.1 was a gift from David Rubinsztein (81)
mCherry-Sequestosome1-N-18	Addgene	#55132	mCherry-Sequestosome1-N-18 was a gift from Michael Davidson
YFP-Sp100A(K297A)-C1	Addgene	#134552	YFP-Sp100A(K297A)-C1 was a gift from Susan Janicki (82)
GFP-Rab27A	Addgene	#89237	GFP-Rab27A was a gift from William Gahl (83)
GFP-Rab27B	Addgene	#89447	GFP-Rab27B was a gift from Wendy Westbroek (84)
pBI-eGFP-Rab31	Addgene	#200265	pBI-eGFP-Rab31 was a gift from Guangpu Li (85)
EGFP-Rab33bb in pCSDEST2	Addgene	#140882	EGFP-Rab33bb in pCSDEST2 was a gift from Rob Parton (86)
pLenti CMV GFP Puro	Addgene	#17448	pLenti CMV GFP Puro (658-5) was a gift from Eric Campeau & Paul Kaufman (87)
pCMV-VSV-G	Addgene	#8454	pCMV-VSV-G was a gift from Bob Weinberg (88)
pCMV-dR8.2 dvpr	Addgene	#8455	pCMV-dR8.2 dvpr was a gift from Bob Weinberg (88)
pcDNA-CIN85-Flag	–^*a*^	–	pcDNA-CIN85-Flag was a gift from Ivan Dikic (89)

^*a*^ “–” indicates that the pcDNA-CIN85-Flag plasmid was a gift.

### Immunoblot analysis

Cells were solubilized in triple detergent buffer (50 mM Tris-HCl [pH 8], 150 mM NaCl, 0.1% sodium dodecyl sulfate, 1% Nonidet P-40, 0.5% sodium deoxycholate, 100 mg/mL of phenylmethylsulfonyl fluoride) supplemented with phosphatase inhibitors (10 mM NaF, 10 mM b-glycerophosphate, 0.1 mM sodium vanadate) and protease inhibitor cocktail (Sigma) and briefly sonicated. Protein concentration was determined with the Bradford method (Bio-Rad Laboratories). The antibodies used along with their dilutions are listed in Table 2. Proteins were visualized with 5-bromo-4-chloro-3 indolylphosphate (BCIP)-nitroblue tetrazolium (NBT) (VWR) or with ECL western blotting detection reagents (Amersham Biosciences).

**TABLE 2:** List of antibodies used

Antibody	Origin	Dilution	Vendor	Catalog #
ICP0	Mouse	1:1,000	Santa Cruz	sc-53070
ICP4	Mouse	1:1,000	Santa Cruz	sc-69809
VP16	Mouse	1:1,000	Santa Cruz	sc-7545
VP22	Mouse	1:1,000	Invitrogen	MA5-16284
gC	Mouse	1:1,000	Invitrogen	MA1-19267
gD	Mouse	1:1,000	Santa Cruz	sc-21719
gB	Mouse	1:1,000	Santa Cruz	Sc-56987
CD63	Mouse	1:1,000	Santa Cruz	sc-5275
Rab5	Mouse	1:1,000	Santa Cruz	sc-46692
ATG5	Mouse	1:1,000	Santa Cruz	sc-133158
Rab7	Mouse	1:1,000	Santa Cruz	sc-376362
TSG101	Mouse	1:1,000	Santa Cruz	sc-7964
EGFR	Mouse	1:1,000	Santa Cruz	sc-373746
CIN85	Mouse	1:1,000	Santa Cruz	sc-166862
CD81	Mouse	1:1,000	Santa Cruz	sc-166029
β-Actin	Mouse	1:1,000	Santa Cruz	sc-47778
β-Actin	Mouse	1:1,000	Sigma-Aldrich	A4700
Flag epitope (M2)	Mouse	1:1,000	Sigma-Aldrich	F1804
LC3B	Rabbit	1:2,000	NOVUS	NB100-2220
STING	Mouse	1:1,000	R&D systems	MAB7169
CIN85	Rabbit	1:1,000	Cell Signaling	12304
p62/SQSTM1	Mouse	1:1,000	Cell Signaling	88588
ALIX	Mouse	1:1000	Cell Signaling	2171
Annexin A1	Rabbit	1:1,000	Fisher Scientific	50-173-3672
ARF6	Mouse	1:1,000	Sigma Aldrich	A5230
Anti-Mouse IgG	Goat	1:2,000	Sigma Aldrich	A4416
Anti-Rabbit IgG	Goat	1:2,000	Sigma Aldrich	A0545
Sp100	Rabbit	1:1,000	GeneTex	GTX131569
Alexa Fluor 594 goat anti-rabbit IgG	Goat	1:1,000	Invitrogen	A11012
Alexa Fluor 594 goat anti-mouse IgG	Goat	1:1,000	Invitrogen	A11005
Alexa Fluor 488 goat anti-rabbit IgG	Goat	1:1,000	Invitrogen	A11008
Alexa Fluor 488 goat anti-mouse IgG	Goat	1:1,000	Invitrogen	A11001

### Immunofluorescence analysis

The procedures were described elsewhere (16). Briefly, the cells were fixed in 4% paraformaldehyde, permeabilized, blocked with PBS–TBH solution consisting of 0.1% Triton X-100 in PBS, 10% horse serum, and 1% BSA, and reacted with primary antibodies (Table 2) diluted in PBS–TBH. The cultures were rinsed several times with PBS–TBH and reacted with appropriate Alexa-Fluor-conjugated secondary antibodies (Table 2), diluted 1:1,000 in PBS–TBH. After several rinses, first with PBS–TBH and then with PBS, the samples were mounted in Vectashield medium (plain or with DAPI) and examined with either a Nikon TI2-E equipped with CSU-W1 SoRa (60× oil ID 1.42 NA objective) or a Leica TCS SP8 STED.

### DAPRed autophagy stain assay

Vero cells were seeded at 70–80% confluency on 4-well slides. Cells were transfected with a CIN85-Flag plasmid using Lipofectamine 3000 Transfection kit (Thermo Fisher Scientific) and then 24 h later were either treated with rapamycin at 5 µM or infected with HSV-1 (F) (10 PFU/cell). At 12 h post infection, DAPRed stain was added for 30 min (0.1 µmol), washed twice with PBS, and re-incubated in 10% DMEM for 4 h. Cells were then fixed using 4% paraformaldehyde, blocked in PBS-TBH (0.1% Triton X-100, 10% horse serum, and 1% BSA in PBS) and then incubated in primary and secondary antibodies for 2 h, respectively. Cells were mounted using Vectashield mounting medium with DAPI and imaged using a Leica TCS SP8 STED.

### Immunoprecipitation analysis

Approximately 5 × 10^6^ hTERT-HEL cells mock infected or exposed to HSV-1(F) (1 PFU/cell) were harvested at 16 h post-infection and resuspended in lysis buffer composed of 50 mM HEPES (pH 7.4), 150 mM NaCl, 1% NP-40, 1 mM EDTA, 1 mM EGTA, 1 mM PMSF, 0.5% sodium deoxycholate, 1 mM NaF, 1 mM Na_3_VO_4_, and protease cocktail inhibitors (Sigma). The cells were incubated for 30 min on ice, cell debris were removed by centrifugation at 8,000 rpm for 10 min, and the supernatant was supplemented with 1 µg CIN85 (D1A4) rabbit monoclonal antibody or an isotype control IgG. After overnight incubation at 4°C, protein A beads (Sigma-Aldrich) were added to the samples and incubation continued for 2 h. Finally, the beads were collected and rinsed four times with the lysis buffer. The immunocomplexes bound on beads were dissolved in loading buffer composed of 4% SDS, 100 mM Tris-Cl (pH 6.8), 20% glycerol, and 0.2% bromophenol blue, supplemented with β-mercaptoethanol, and boiled for 5 min. After centrifugation to remove the beads, the eluted proteins were electrophoretically separated in denaturing polyacrylamide gels and analyzed by western blot.

### Extracellular vesicle purification

Isolation of EVs from infected or uninfected cells was done as previously described with minor modifications (30, 57). Briefly, hTERT-HEL cells were infected with HSV-1(F), the ICP0 mutants (0.5 PFU/cell), or remained uninfected. The supernatant was collected at 48 h post-infection, centrifuged at 1,000 × *g* for 5 min and at 2,000 × *g* for 20 min, and filtered through a 0.45-μm-pore-size filter. Then, the supernatant was concentrated (molecular mass cutoff, 100 kDa; Centricon Plus 70) according to the manufacturer’s instructions (Millipore). The supernatant was loaded on top of an iodixanol-sucrose gradient that ranged from 6% to 18% with a 1.2% increment. The 60% iodixanol was diluted in 10 mM Tris, pH 8, and 0.25 M sucrose. Samples were centrifuged in an SW41Ti rotor for 2 h at *r*_max_250,000 × *g* at 4°C in a Beckman Coulter Optima XPN-80 ultracentrifuge. Fractions (500 µL) were collected from the top to the bottom of the gradient for further analysis.

### Nanoparticle tracking analysis

NTA was performed in the fractions of the gradient that contained EVs using the NanoSight LM10 instrument (NanoSight, Salisbury, United Kingdom). For each sample, nine different acquisitions were obtained of 60 s each. NTA software version 2.3 was used to analyze 60 s videos of data collection to obtain the mean, median, and mode of vesicle size and concentration.

### Statistical analysis

The *P* values were calculated using a two-tailed Student’s *t*-test with a *P* ≤ 0.05 considered significant or a two-way ANOVA with Tukey’s post hoc. Stars represent the following values: *P ≤ 0.05, **P ≤ 0.01, and ***P ≤ 0.001. All statistical analyses were performed using at least three biological replicates to ensure reproducibility. The Pearson’s correlation coefficient was calculated for the vesicular structures using the Image J and the JACoP (Just Another Co-localization) plugin.

## SUPPLEMENTAL MATERIAL

Figure S1 mbio.02143-25-s0001.tif Growth defects of ICP0 delta244-277 virus are not due to deficiencies in the ICP0 E3 ubiquitin ligase activity.

Figure S2 mbio.02143-25-s0002.tif Localization of vesicular markers in uninfected cells.

Figure S3 mbio.02143-25-s0003.tif The ICP0/CIN85 vesicles do not colocalize with Rab31 or Rab33bb and do not stain with DAPRed.

Figure S4 mbio.02143-25-s0004.tif Some ICP0/CIN85 vesicles colocalize with Rab27a and Rab27b.

Figure S5 mbio.02143-25-s0005.tif Colocalization of ICP0 and CIN85 with different vesicular markers in infected and uninfected cells.

Supplemental text mbio.02143-25-s0006.docx Legends for Fig. S1-S5.

ASM does not own the copyrights to Supplemental Material that may be linked to, or accessed through, an article. The authors have granted ASM a non-exclusive, world-wide license to publish the Supplemental Material files. Please contact the corresponding author directly for reuse.
