# Supplementary figures and images for: Herpes simplex virus diverts CIN85 endosomal cargo for exocytosis to evade antiviral responses: a novel role for the viral immediate-early protein ICP0
Source: mBio. 2025 Sep 24;16(11):e02143-25. doi: 10.1128/mbio.02143-25 (PMC12607867; doi:10.1128/mbio.02143-25)

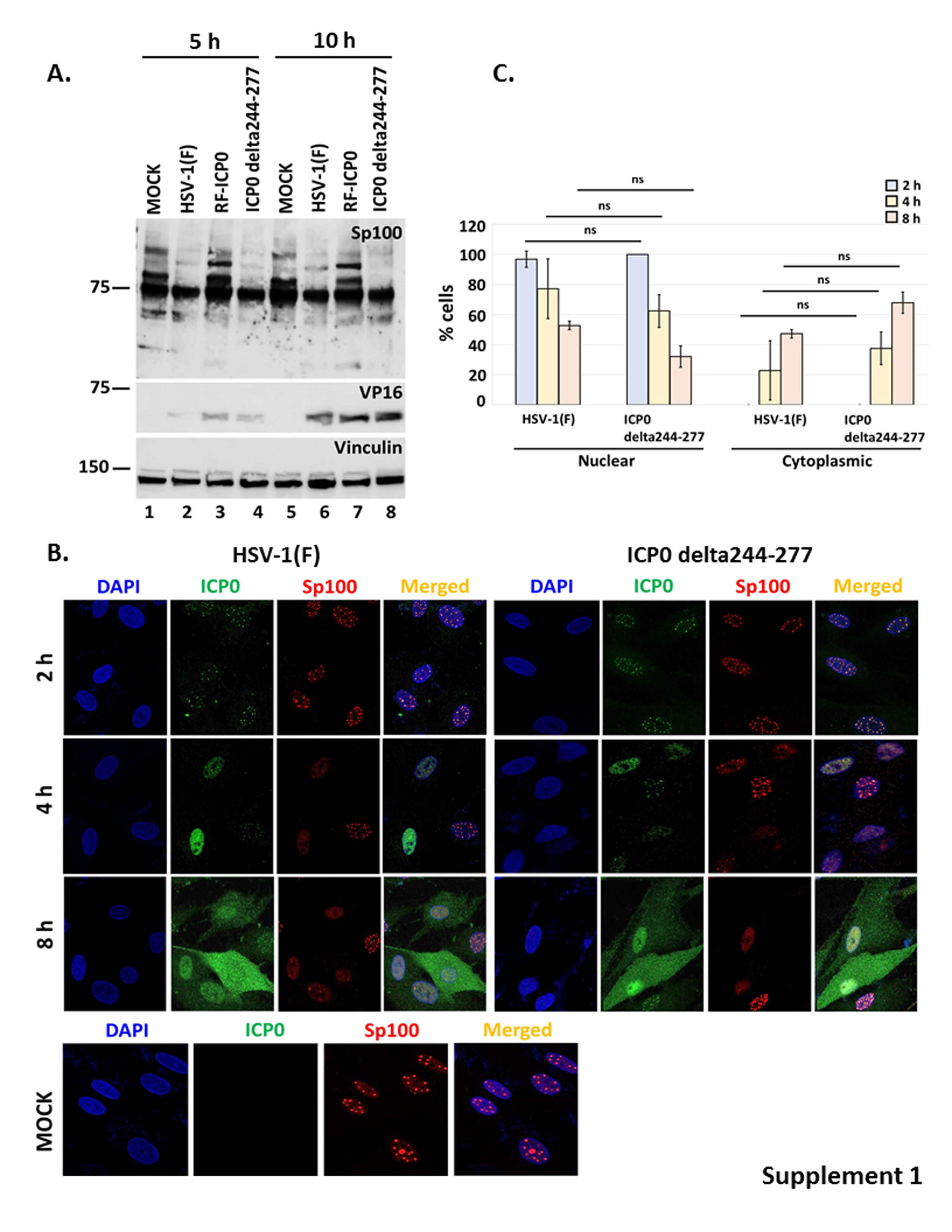

Supplement: Figure S1 — Growth defects of ICP0 delta244-277 virus are not due to deficiencies in the ICP0 E3 ubiquitin ligase activity. [file mbio.02143-25-s0001.tif]

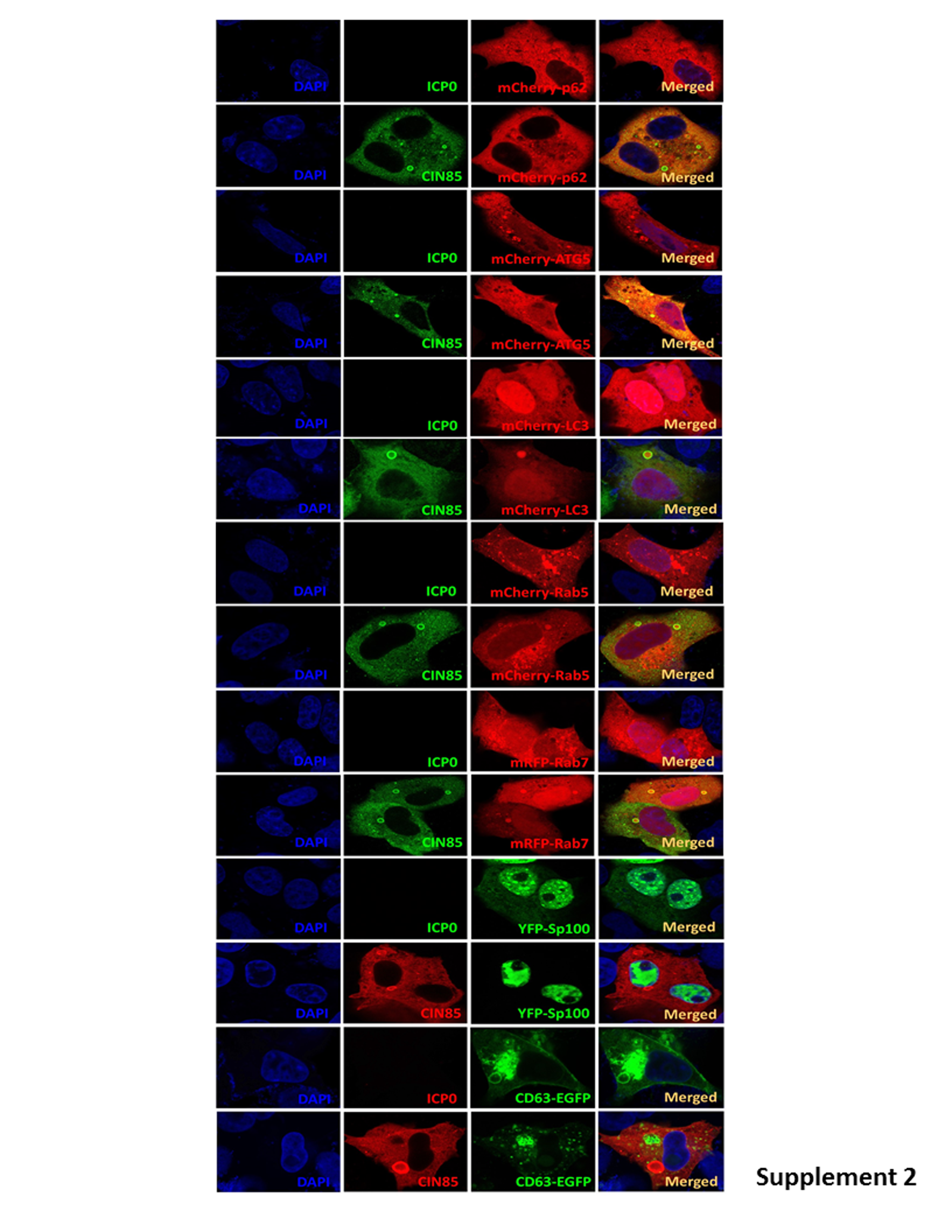

Supplement: Figure S2 — Localization of vesicular markers in uninfected cells. [file mbio.02143-25-s0002.tif]

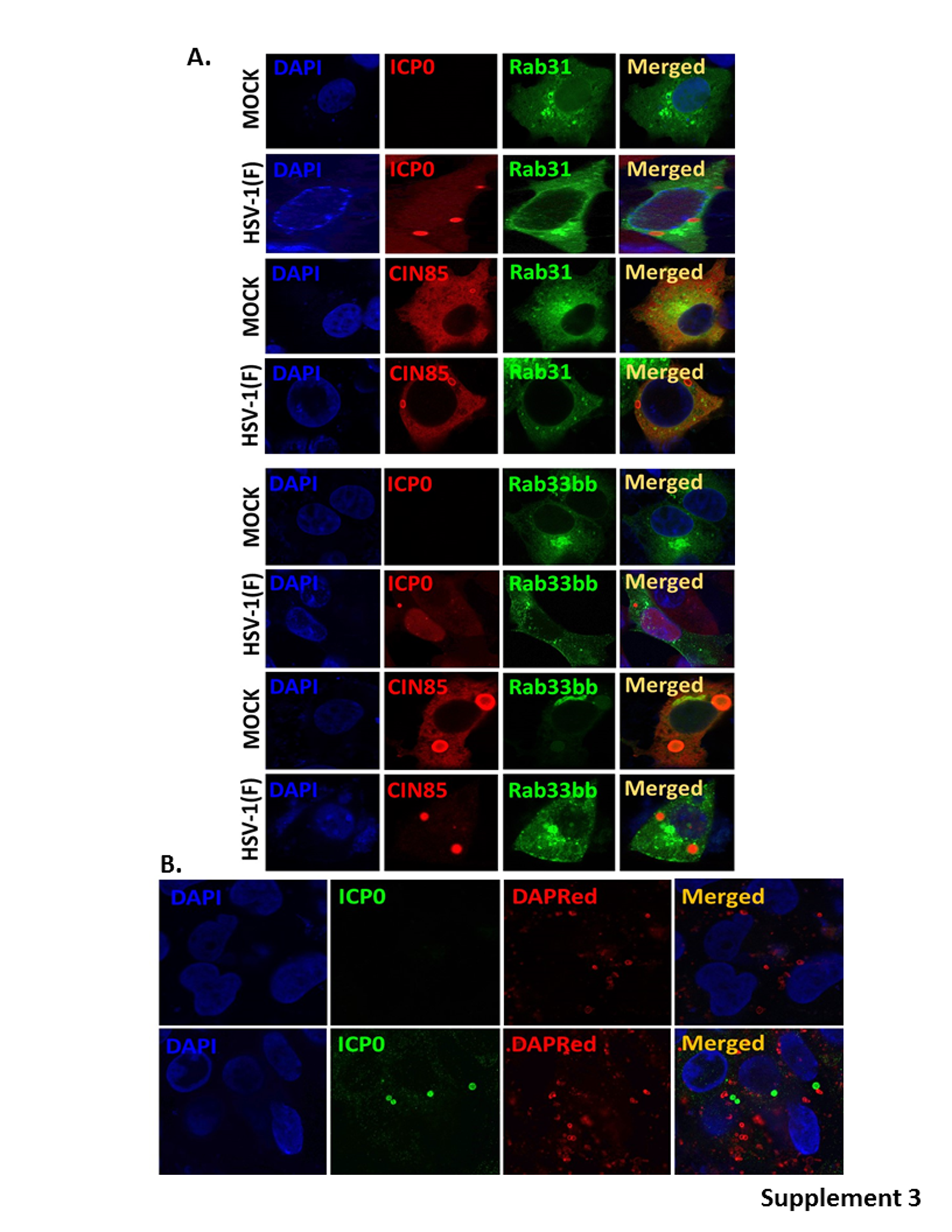

Supplement: Figure S3 — The ICP0/CIN85 vesicles do not colocalize with Rab31 or Rab33bb and do not stain with DAPRed. [file mbio.02143-25-s0003.tif]

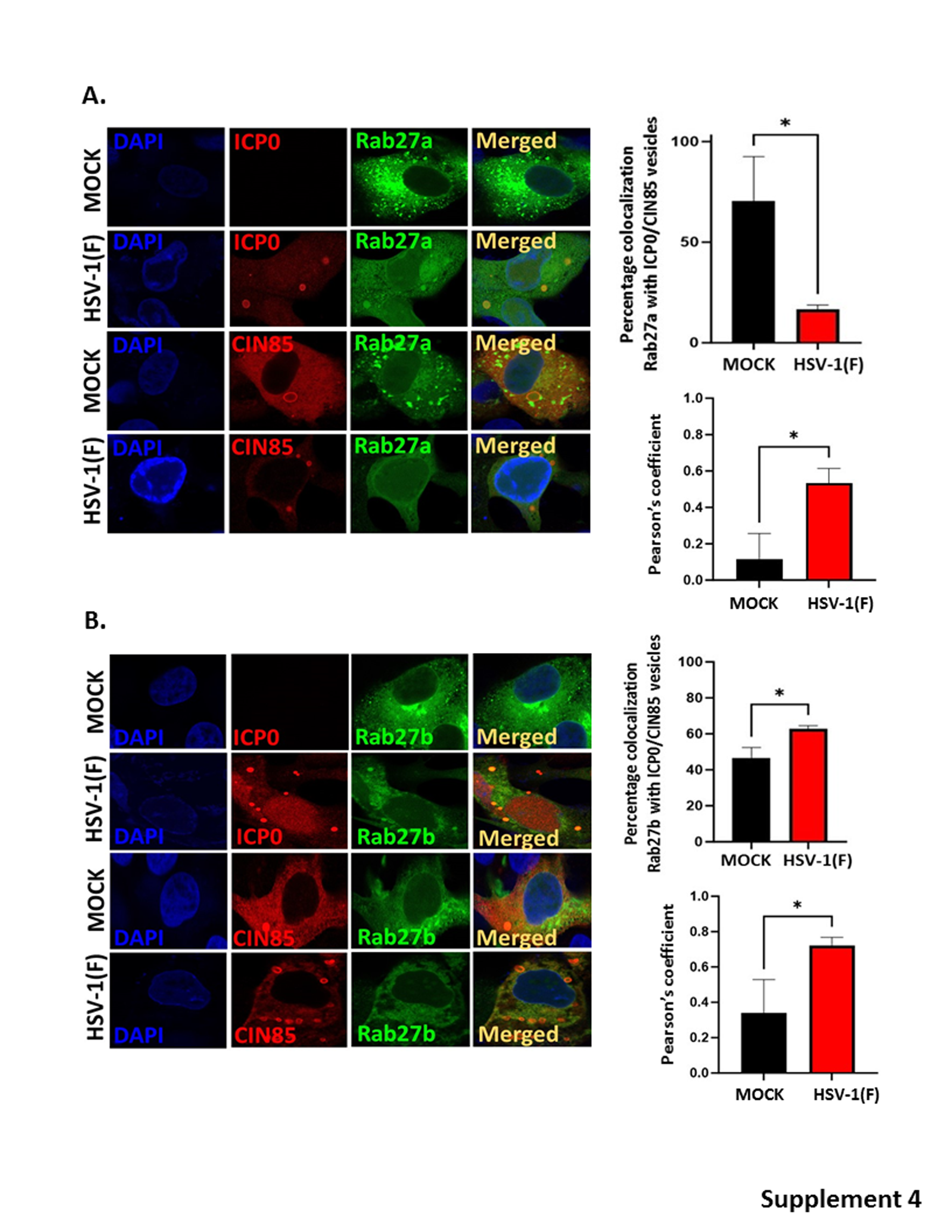

Supplement: Figure S4 — Some ICP0/CIN85 vesicles colocalize with Rab27a and Rab27b. [file mbio.02143-25-s0004.tif]

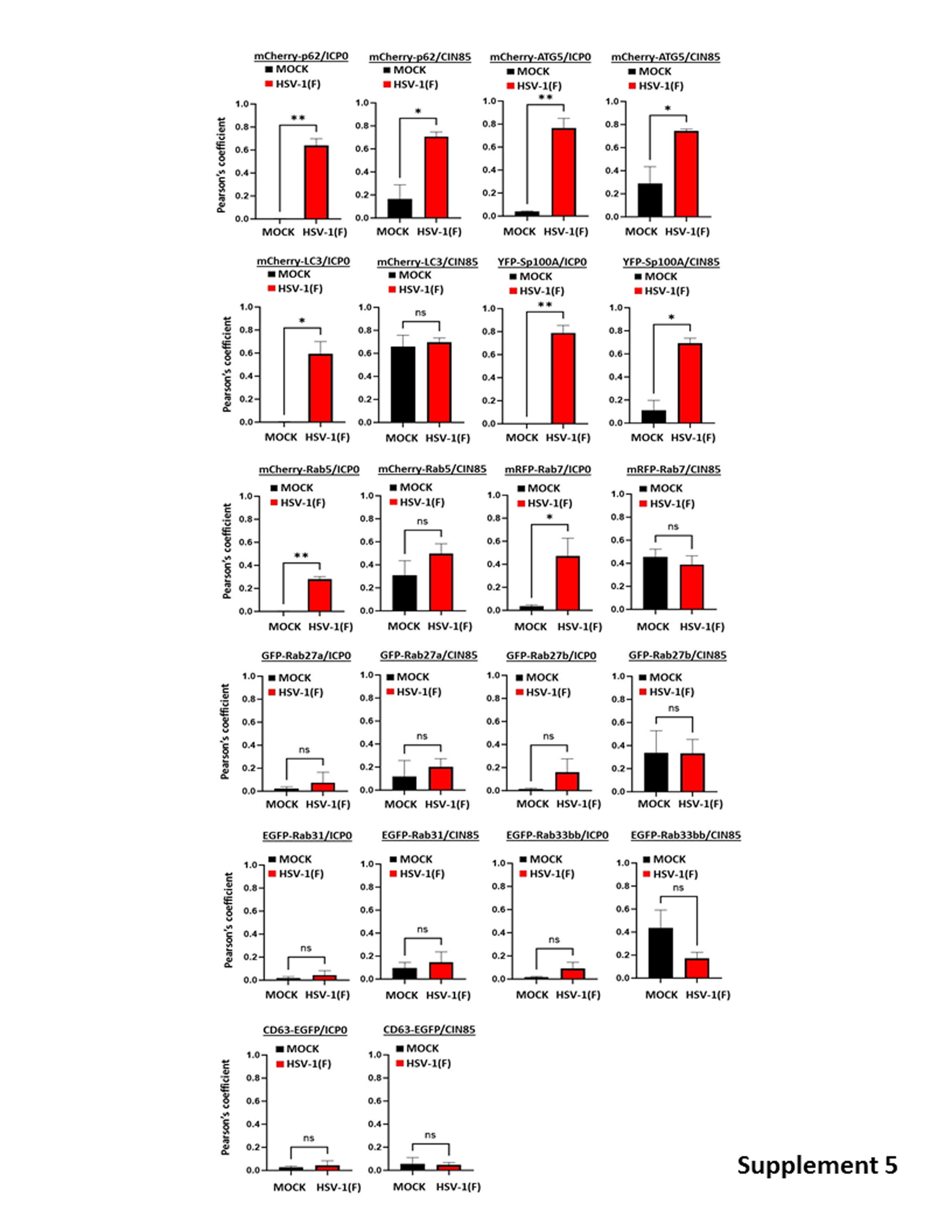

Supplement: Figure S5 — Colocalization of ICP0 and CIN85 with different vesicular markers in infected and uninfected cells. [file mbio.02143-25-s0005.tif]
