# Supplementary material for: Herpes simplex virus diverts CIN85 endosomal cargo for exocytosis to evade antiviral responses: a novel role for the viral immediate-early protein ICP0
Source: mBio. 2025 Sep 24;16(11):e02143-25. doi: 10.1128/mbio.02143-25 (PMC12607867; doi:10.1128/mbio.02143-25)
Supplement: Supplemental text — Legends for Fig. S1-S5. [file mbio.02143-25-s0006.docx]

**Supplemental information**

**Figure S1: Growth defects of ICP0 delta244-277 virus are not due to deficiencies in the ICP0 E3 ubiquitin ligase activity. A.** hTERT-HEL cells were uninfected, infected with HSV-1(F), RF-ICP0, and ICP0 ΔCIN85exo virus (10 PFU/cell). The cells were harvested at 5 and 10 h post-infection and equal amounts of proteins from total cell lysates were analyzed for Sp100. VP16 served as a control for infection and vinculin as a loading control. **B.** hTERT-HEL cells were infected with HSV-1(F) or ICP0 delta244-277 virus (10 PFU/cell). The cells were fixed at 2, 4, and 8 h post-infection and doubly reacted with a Sp100 and an ICP0 antibody. Images were captured with the same settings of a Leica TCS SP8 STED microscope. **C.** Quantification of cells with nuclear versus cytoplasmic ICP0 from cultures infected with either the WT or the ICP0 delta244-277 virus as in panel B. Images were acquired randomly.

**Figure S2:** **Localization of vesicular markers in uninfected cells.** Vero cells were transfected with mCherry-p62, mCherry-ATG5, mCherry-LC3, mCherry-Rab5, mRFP-Rab7, YFP-Sp100A, CD63-EGFP followed by HSV-1(F) infection (10 PFU/cell) at 24 h post-transfection. The cells were fixed at 14 h post-infection and stained with an ICP0 antibody and DAPI. Images were captured using a TCS SP8 STED microscope. Replicate cultures of Vero cells were also co-transfected with the above-mentioned plasmids and a Flag-CIN85 –expressing plasmid. At 24 h post-transfection the cells were stained an anti-CIN85 antibody and DAPI. Images were obtained as above.

**Figure S3: The ICP0/CIN85 vesicles do not colocalize with Rab31 or Rab33bb and do not stain with DAPRed.** **A.** Vero cells were transfected with plasmids expressing EGFP-Rab31, EGFP-Rab33bb, or co-transfected with a Flag-CIN85–expressing plasmid and plasmids expressing the abovementioned proteins. At 24 h post-transfection the cells were infected with HSV-1(F) (10 PFU/cell) or remained uninfected. Cells were fixed at 14 h post-infection and probed with an ICP0 or a CIN85 antibody. Images were captured using a TCS SP8 STED microscope. **B.** Vero cells were transfected with a Flag-CIN85-expressing plasmid and at 24 h post-transfection the cells were either infected with HSV-1(F) (10 PFU/cell) or remained uninfected. At 12 h post infection DAPRed stain was added as indicated in the materials and methods. Cells were then fixed and probed with ICP0 antibody. Images were captured using a TCS SP8 STED microscope.

**Figure S4: Some ICP0/CIN85 vesicles colocalize with Rab27a and Rab27b. A-B.** Vero cells were transfected with plasmids expressing GFP-Rab27a, GFP-Rab27b, or co-transfected with a Flag-CIN85–expressing plasmid and plasmids expressing the abovementioned proteins. At 24 h post-transfection the cells were infected with HSV-1(F) (10 PFU/cell) or remained uninfected. Cells were fixed at 14 h post-infection and probed with an ICP0 or a CIN85 antibody. Images were captured using a TCS SP8 STED microscope. ICP0/CIN85 vesicles colocalizing with either Rab27a or Rab27b were quantified from randomly acquired images. Pearson’s correlation coefficient was calculated for ICP0/CIN85 vesicles colocalizing with these Rabs. For vesicles that did not appear to colocalize with these Rabs the Pearson’s correlation coefficient was calculated independently and is depicted in Figure S5.

**Figure S5: Colocalization of ICP0 and CIN85 with different vesicular markers in infected and uninfected cells.** Pearson’s correlation coefficient was used to determine the extent of colocalization of ICP0 and CIN85 with all the vesicular markers discussed in Figure 3, Figure S2, and Figure S3 in uninfected and HSV-1 infected cells. Colocalization was quantified only in the vesicular structures.
